# Biowaste to biochar: a techno-economic and life cycle assessment of biochar production from food-waste digestate and its agricultural field application

**DOI:** 10.1007/s42773-025-00456-0

**Published:** 2025-03-10

**Authors:** Disni Gamaralalage, Sarah Rodgers, Andrew Gill, Will Meredith, Tom Bott, Helen West, Jessica Alce, Colin Snape, Jon McKechnie

**Affiliations:** 1 Sustainable Process Technologies Research Group, Faculty of Engineering, The University of Nottingham, Nottingham, NG7 2RD UK; 2 R&D Manager, Invica Industries Ltd, The University of Nottingham, Energy Technologies Building, Jubilee Campus, Nottingham, NG7 2TU UK; 3 Faculty of Engineering, The University of Nottingham, Energy Technologies Building, Jubilee Campus, Nottingham, NG7 2TU UK; 4 School of Biosciences, Sutton Bonington Campus, The University of Nottingham, Leicestershire, LE12 5RD UK; 5 Strategy & Development, Severn Trent Green Power, The Stables, Radford, Chipping Norton, OX7 4EB UK

**Keywords:** Biochar, Anaerobic digestate, Food waste, Life cycle assessment, Techno-economic assessment, Greenhouse gas removal

## Abstract

Biochar has high potential for long-term atmospheric carbon storage in terrestrial environments, contributing to meeting the UK and global greenhouse gas emission reduction targets. This study investigates the greenhouse gas emissions and techno-economics associated with biochar produced from food waste anaerobic digestate using hydrothermal carbonisation followed by high-temperature post carbonisation. Owing to high moisture contents, digestates are challenging to valorise. However, these low-value feedstocks have steady availability with minimal competition for other applications. The study focuses on food waste digestate supply, biochar production, biochar agricultural field application, and transportation activities. Minimising digestate transport through co-locating biochar production facilities with anaerobic digestion displayed greenhouse gas mitigation costs of < £100 tCO_2_eq^−1^ (125 USD tCO_2_eq^−1^). The 88% stable carbon fraction of the biochar, which is resistant to degradation in soil, is primarily responsible for the effective removal of atmospheric greenhouse gases. This results in net emissions reductions of 1.15–1.20 tCO_2_eq per tonne of biochar, predominantly due to the long-term storage of durable carbon (1.7 tCO_2_eq per tonne of biochar). Using 50% of the UK’s projected available food waste digestate by 2030 offers a sequester potential of 93 ktCO_2_eq p.a., requiring 28 biochar facilities at 20 kt p.a. capacity. Sensitivity analysis emphasises the influence of the gate fee charged to process digestate, highlighting its importance for economic success of the biochar production. Further studies are needed to investigate the potential technology enhancements to reduce fossil-fuel use and provide greater certainty of the co-benefits of biochar application in agricultural soil.

**Graphical Abstract:**

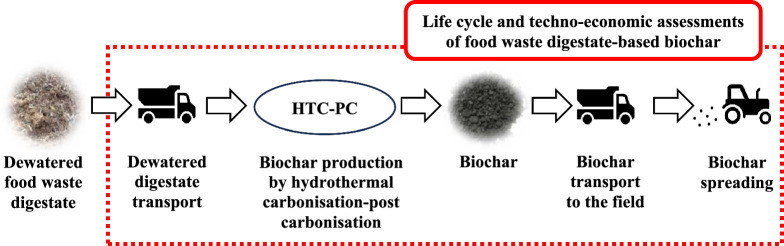

**Supplementary Information:**

The online version contains supplementary material available at 10.1007/s42773-025-00456-0.

## Article Highlights

- Plastic contamination makes food waste digestate expensive to manage.
- Hydrothermal carbonisation combined with post carbonisation is an efficient route to producing stable biochar.
- Distributed biochar production sequesters carbon for less than £100 per tCO_2_eq.

**Supplementary Information:**

The online version contains supplementary material available at 10.1007/s42773-025-00456-0.

## Introduction

Cost-effective carbon dioxide removal (CDR) technologies are essential to meet greenhouse gas (GHG) emissions targets, such as the UK’s net zero target by 2050 (Simon et al. 2021). Stable biochar achieves long-term carbon storage by transferring atmospheric CO_2_ to terrestrial stocks along with potential co-benefits for soil quality (Ding et al. 2016; Oni et al. 2019). However, steady availability of suitable low-cost feedstocks for biochar production is challenging. Utilising wet biogenic wastes with less desirability in conventional energy applications is strategic to avoid significant financial and environmental costs in existing waste managements. Employing biowaste-to-biochar schemes through utilising digestate from anaerobic digestion (AD) could extend the feedstock range for biochar production, provided significant net GHG removals are achieved at acceptable costs.

Food waste (FW) is a global challenge, representing wasted use of resources (land, water, mineral, energy) and waste management burdens. The collection and separation of organic wastes, including FW, in the UK is undergoing significant reform aimed at standardizing waste collection systems and ensuring FW is managed separately from general and recyclable waste. This approach will help reduce contamination in recyclable materials and enhance waste treatment processes, such as AD (Department for Environment 2020; ISO Compliance Register 2024). Landfilling FW results in intensive landfill gas emissions, and important policy incentives aim to divert FW’s destination, e.g., the UK landfill tax standard rate: £98.6 t^–1^ (GOV.UK 2022). The UK’s 25-year environment plan targets to eliminate avoidable waste by 2050 and FW from landfill by 2030 (DEFRA 2018). As such, AD is increasingly employed to manage FW in the UK (Victor and Moves 2020; WRAP 2022). However, plastic packaging contamination in the produced digestate prohibits its direct deposition on agricultural fields owing to concerns over cost, use, and management (Environment Agency 2014; Xiao et al. 2022). As a result, FW digestate is often incinerated in the UK at high costs (WRAP 2021), forgoing the opportunity to recycle nutrients to agricultural soils. Furthermore, digestate incineration yields a minimal net energy recovery due to its high moisture content. To accommodate increasing FW digestate production over the coming years, effective routes for FW digestate management addressing cost and environmental burdens of current disposal methods are essential. Comparatively, the regulations concerning biochar use in agriculture in the UK are notably stricter compared to the new EU Fertiliser Regulation. While biochar from waste streams is a developing area, concerns around contamination, heavy metals, and organic pollutants limit its broader acceptance under UK standards. Current frameworks, such as BSI PAS100 and the UK Biochar Quality Mandate, primarily focus on ensuring that biochar is safe, stable, and beneficial for soil application, but exclude waste-based feedstocks for this purpose (Houses of Parliament 2010; Shackley et al. 2014). However, the EU Fertiliser Regulation permits biochar in fertilising products, provided it meets stringent criteria for contaminants and stability, with some restrictions on feedstock source (European Parliament and Council of European Union 2019). This regulatory shift aims to promote circular economy principles by utilizing waste-derived materials more effectively while maintaining environmental safety standards (Directorate-General for Internal Market, Industry 2022).

Biowastes with high moisture contents (e.g., FW digestate) are incompatible for solid fuel production or direct carbonisation to biochar due to excessive pre-treatment (drying) costs. Hydrothermal carbonisation (HTC) is an emerging technology that treats wet biowaste at lower temperatures than pyrolysis (Kumar and Ankaram 2019; Yoganandham et al. 2020). Performing HTC with post carbonisation (HTC-PC) produces stable biochar with low proportions of degradable carbon (Stirling et al. 2018; Musa et al. 2022). HTC is yet to be deployed commercially due to uncertainty surrounding financial viability, energy efficiency, and achievable net GHG reduction. A combined life cycle and techno-economic assessment (LCA-TEA) would enable the comprehensive evaluation of biochar production from FW digestate, providing an understanding of the trade-offs between technical, economic, and environmental performance metrics (Mahmud et al. 2021).

Numerous LCA and TEA studies have assessed CDR technologies, highlighting their potential for CO₂ reduction (Goglio et al. 2020; Terlouw et al. 2021; Asibor et al. 2021). In the context of biochar production, most research has focused on biochar derived from woody biomass, typically produced via pyrolysis (Matuštík et al. 2020; Lefebvre et al. 2021; Marzeddu et al. 2021). Marzeddu et al. (2021) demonstrated a GHG emission of over − 8 tonnes of CO_2_ per tonne of biochar produced from woodchips, while Lefebvre et al. (2021) reported − 1.6 tonnes of CO_2_ per tonne of biochar GHG emission produced from sugarcane residue. Matustik et al. (2020) reported that GHG emissions from biochar systems can range from − 2.5 tonnes of CO_2_ per tonne of biochar in optimized systems characterized by efficient pyrolysis and effective carbon sequestration to approximately 0.12 tonne CO_2_ per tonne of biochar in less efficient scenarios, where biochar stability is low, and energy recovery is minimal or poorly implemented. These findings underscore the significant CO_2_ removal potential of biochar, which varies depending on the feedstock used. However, biochar production from dedicated energy crops presents additional challenges, primarily due to the high GHG emissions and costs associated with crop cultivation (Matuštík et al. 2020; Deng et al. 2024). Deng et al. (2024) reported higher negative emission costs for biochar derived from dedicated crops, ranging from 101 to 144 USD per tonne of CO_2_ removed, compared to the lower negative emission costs for biochar from agricultural residues, which range from 60 to 96 USD per tonne of CO_2_ removed. While agricultural residues offer a lower-emission alternative, their use often competes with energy applications, such as bioenergy (Lefebvre et al. 2021). In contrast, digestate, a byproduct of AD, represents a largely untapped yet promising feedstock for biochar production. While existing studies on FW and digestate-based biochar have primarily focused on applications such as fertilizer (Hung et al. 2017; Song et al. 2021), additive in AD (Cavali et al. 2022) or construction material (Chen et al. 2024), the potential of digestate-derived biochar as a CDR technology remains underexplored. This study addresses this critical gap by quantifying the financial costs and GHG emissions associated with producing biochar from FW digestate. It evaluates both the current and future potential of digestate-derived biochar as a viable CDR solution in the UK, offering insights with global applicability. The findings will provide valuable insights for advancing biochar frameworks and carbon offset schemes, contributing significantly to sustainable waste management and climate change mitigation. This work is particularly relevant to the scientific community, offering actionable data that can guide the integration of FW digestate into global CDR strategies.

## Materials and methods

In this study, experimental investigations and modelling were combined to assess the viability of biochar production from FW digestate as a CDR technology. Experimental activities are described in Section S1 in the Supplementary Information (SI). Rigorous LCA and TEA models are detailed in Sects. 2.1 and 2.2, respectively. These models were developed using Microsoft Excel and are underpinned by a comprehensive database constructed from a combination of primary and secondary data sources. Primary data were collected directly from lab-scale and demonstration facility trials, while secondary data were obtained from government publications (e.g., WRAP, DESNZ), commercial databases (e.g., ecoinvent), and previous peer-reviewed publications. Where primary data were unavailable, reasonable assumptions were made based on peer-reviewed literature and guidance from industrial partners.

The inventory data used in this study are presented in Table 1, while additional parameters are provided in Table S1 (DEFRA 2013, 2020; BEIS 2017; WRAP 2018, 2021; Szwaja et al. 2019; DESNZ 2023a, b) of the SI. The developed models were employed to evaluate the net GHG removal potential, biochar production cost, and overall levelized cost of carbon sequestration. To ensure consistency, the Excel-based LCA-TEA models were validated by comparing their outputs with results from peer-reviewed LCA and TEA studies. Additionally, sensitivity analyses were performed to confirm the robustness of the results.

**Table 1:** Inventory data for production and application of 1 tonne of biochar in agriculture soil

Inventory	Value	Unit
FW digestate		
FW input for AD facility	70.77	tonnes
Digestate quantity	10.26	tonnes
Carbon in dry digestate (0% moisture content)	1.25	tonnes
HTC-PC		
Natural gas use	1.48	MWh
Grid electricity	0.51	MWh
Process water from HTC-PC	1.74	tonnes
Biochar yield	1.00	tonnes
Carbon content in biochar	0.53	tonnes
Operating cost	307.69	£ p.a
Annualised CAPEX	389.74	£ p.a
Biochar soil application		
Biochar transport cost	0.22	£ tkm^−1^
Biochar spreading cost^a^	3.50	£

^a^(WRAP 2016)

### Life cycle GHG emissions and net GHG removals

**Fig. 1:**
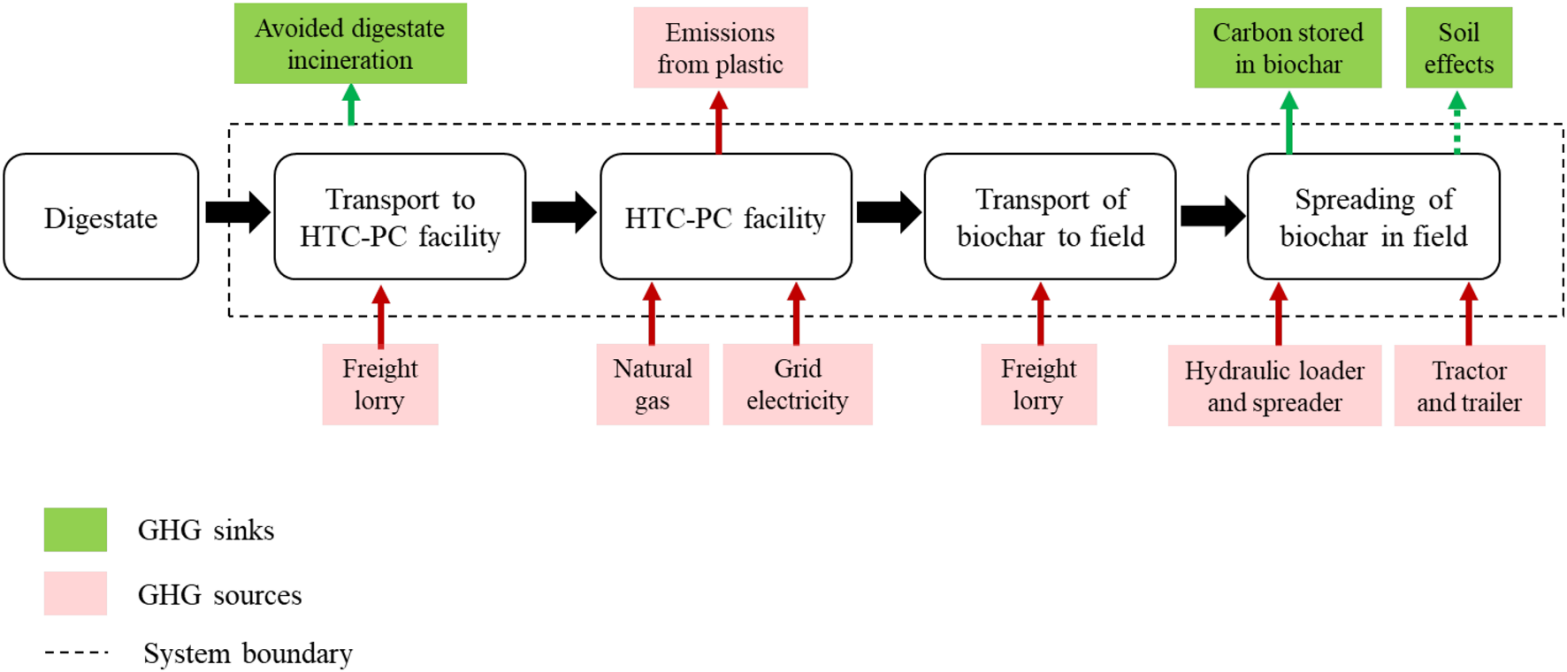
System boundary of the study

LCA models were developed to evaluate biochar production from FW digestate and its application on UK agricultural lands. The system boundary includes activities from FW digestate supply (including avoided incineration), biochar production by HTC-PC, all relevant transportation stages, and application of biochar to agricultural fields (Fig. 1). Several scenarios for commercial deployment of biochar production in 2030 were analysed considering different aspects within the system boundary.

- Scenario 1) Base case: Distributed HTC-PC facilities co-located with AD facilities, avoiding the need to transport digestate. Excluding fertilizer displacement by biochar application.
- Scenario 2) Digestate transport case: Centralised HTC-PC facilities requiring the digestate to be transported between AD and HTC-PC facilities. Excluding fertilizer displacement by biochar application.
- Scenario 3) Soil effect case: Scenario 1 including fertilizer displacement, assuming nitrogen/phosphorous/potassium (NPK) content in the labile biochar fraction is available for crops.

Net GHG emissions within the system boundary of biochar production process were evaluated considering CH_4_, N_2_O and CO_2_ emissions which are converted to CO_2_ equivalents (CO_2_eq) with the 100-year time horizon IPCC global warming potential values (Forster et al. 2021). The required biochar application rates to obtain its benefits on soil are site- or crop-specific (Gao et al. 2021; Li et al. 2022). As such, the functional unit in this study was taken as the production and application of one tonne of biochar in agricultural soil. Details on the HTC-PC biochar process are provided in SI under Section S2.

#### Food waste digestate supply

The availability of FW digestate was estimated based on FW generation statistics in UK, its application in AD, and considerations of future trends, targets, and expectations. The Waste and Resources Action Programme (WRAP) estimated that in 2021, 10.7 Mt of FW was generated within UK households (6.4 Mt), hospitality & food service (1.1 Mt), food manufacture (1.4 Mt), retail and farm sectors (1.6 Mt) (WRAP 2023a). The predicted FW availability in 2030 was estimated assuming the achievement of the Sustainable Development Goals (SDG) target of halving FW per capita relative to 2007 by 2030 (FAO 2023). This is used as a conservative estimate of FW future availability in the UK. A second FW availability scenario is considered, where the SDG target is not met, and FW generation continues at the current per capita rate. To translate FW availability to FW digestate for biochar production WRAP’s digestate production coefficient (0.87) was used (WRAP 2015; Victor and Moves 2020). Typically, digestate from AD contains around 95% moisture. In this work, it is assumed that the dewatered digestate of 70% moisture is transported to be used for biochar production. Throughout the manuscript, digestate with 70% moisture is referred to simply as ‘digestate’. Current AD capacity in the UK is 4.2 MT for FW with a total capacity of 14 MT (Victor and Moves 2020; DEFRA 2021). Whilst not all FW currently goes to AD, its expansion is expected and is evidenced by the significant increase from 2019 to 2023 (DEFRA 2024).

#### Feedstock and biochar transportation

Scenarios 1 and 3 assume that commercial scale HTC-PC facilities are to be co-located with AD facilities, negating the need for digestate transportation. Scenario 2 considers digestate transport to centralised HTC-PC facilities at a capacity of 20 kt p.a. In all scenarios transportation of biochar to agricultural land for spreading was included. Transportation distances for digestate and biochar were estimated to be 37 km, assuming approximately evenly distributed facilities across the UK. The distances were used as an indicative estimate to determine the impact of transportation. The same distance was assumed for both digestate and biochar transportation. Owing to uncertainty around the actual transportation distance a sensitivity analysis is included to determine the influence of this parameter on the results (see Sect. 3.4). Both digestate and biochar were assumed to be transported by 25 t-capacity lorries.

#### Avoided digestate incineration and plastic content

FW digestate cannot be directly applied to the land owing to its plastic contamination. At present, FW digestates are disposed of via landfilling or incineration, at a cost to the operator, with incineration as the preferred option due to its lower cost compared to landfill (WRAP 2021). Incineration results in the release of the biogenic and fossil-based carbon of the digestate in the plastic content to atmosphere as CO_2_. In contrast, while biochar production results in oxidation of the plastic fraction, a fraction of the biogenic carbon is sequestered in the final biochar product (see 2.1.4 for more detail on the carbon balance of biochar production). Plastic contamination in digestates and FW in the UK has been demonstrated in several studies (Aspray et al. 2018; Aspray and Tompkins 2019; WRAP 2023b). Aspray et al. (2018) reported a range of 340–2500 plastic particles per kg on dry solid bases in digestates collected from Scottish sites (Aspray et al. 2018), while Aspray and Tompkins (2019) demonstrated a 3.4–8.4% fresh weight basis plastics contamination in commercial FWs (Aspray and Tompkins 2019). The 2023 WRAP report summarises the literature on plastics content in UK digestates, demonstrating a value of > 5% dry basis (WRAP 2023b). In this work, a value of 5% (dry basis) has been used based on the aforementioned literature and information from our collaborating industrial partner (Severn Trent Green Power). The carbon content and subsequent emissions from incineration of the plastic content were estimated based on the average composition of collected kerbside plastic (Table S1) and the stoichiometric conversion of the carbon content to CO_2_ emissions. Note that any formation of N_2_O during incineration has been omitted from this analysis as it is assumed that the incineration facility is operated at an appropriate temperature to prevent the formation and ammonia based selective noncatalytic reduction has been implemented (Svoboda et al. 2006). Owing to their biogenic origin, the CO_2_ emissions from the biogenic carbon content in digestate were not included in the direct emissions from incineration.

Notably, incineration of digestate generates renewable electricity. The loss of this renewable electricity generation needs to be accounted for when considering the diversion of digestate to biochar production. The electricity generated by incinerating digestate was estimated using the measured lower heating value of the digestate feedstock, moisture content, and energy conversion efficiency (DEFRA 2013) (Table S1). It was assumed that the electricity from incineration would have displaced grid electricity. Therefore, the emission factor for UK average grid mix was used to account for this forgone renewable electricity production and associated GHG emission that is attributed to biochar production.

#### Biochar production

A facility size of 20 kt p.a. (0.75 t-dry digestate h^−1^) FW digestate at 70% moisture content is considered for all three scenarios. These facilities consist of four HTC reactors and a rotary kiln for PC. The facility size was dictated by the modular nature of HTC reactors, each capable of processing 5 kt p.a. of digestate (0.19 t-dry digestate h^−1^). Therefore, there is limited economic benefit in scaling up facility sizes, and larger facilities necessitate greater digestate transportation, negatively impacting process economics. Considering the typical operating temperatures of HTC (150–300 °C) (Chen et al. 2021) and the carbonisation (500–900 °C) (Nicholas et al. 2019) processes, the HTC process was operated at low temperature (200 °C) and PC at higher temperatures (750 °C) to produce highly stable biochar with lab and demonstration-scale trials conducted in the study (Section S1 in SI for experimental methods). The HTC process produces hydrochar, while the higher temperature PC converts this to stable biochar. Hydrochar exhibits higher O/C and H/C ratios, thus lower aromaticity resulting in poorer stability when added to soil, while biochar demonstrates higher stability (Wang et al. 2021; Suarez et al. 2023) (Sect. 2.1.5).

The process liquid produced during HTC-PC is used internally within the HTC-PC plant, with a residual 170 L t^−1^_digestate_ returned to the AD facility. Recent studies have assessed the impact of process liquids on AD systems. While limited evidence suggests that microorganisms may be affected by certain species in the HTC liquor, both the liquor and biochar have been shown to enhance biomethane yields when added to sewage sludge (Ferrentino et al. 2020). In isolation, adding hydrochar can further increase methane yields in AD (Murillo et al. 2022). Additionally, inoculants, including brewing waste, have been reported to improve biomethane yields (Rubia et al. 2018). Paul et al. (2018) produced biomethane from the HTC liquid phase and achieved an average methane content of 57.7% (Paul et al. 2018) while Aragón-Briceño et al. (2017, 2020) validated the use of process water for biomethane production, obtaining biogas with high methane content (74–80%) (Aragón-Briceño et al. 2017, 2020), Although there may be risks related to toxicity depending on the concentration and composition of the organic compounds, these studies suggest that process liquid can potentially be returned to AD without significantly inhibiting microbial activity.

Importantly, drying of digestate prior to HTC is not required, unlike in thermal processes such as pyrolysis and gasification (Kumar and Ankaram 2019; Nasrollahzadeh et al. 2021). Furthermore the low HTC operating temperature compared to other thermochemical processes (Yoganandham et al. 2020; Nasrollahzadeh et al. 2021) achieves a comparatively energy efficient approach for biochar production from digestate and other high moisture content feedstocks. Section S2 in the SI provides a detailed description of the HTC and PC processes considered in this study, while Fig. S1 presents the process flow diagram (Farthing 2020). Additionally, Fig. S2 illustrates the mass and energy balance of the system.

The inventory data of the biochar production process (Table 1) are based on the existing HTC plant (10 kt p.a. demonstration facility) located at Invica Industries site in Immingham. Energy consumption in the HTC process is primarily influenced by the solids fraction, reaction temperature, and residence time, while being largely independent of the composition of the solid material (Kossińska et al. 2023). Consequently, energy consumption data for the HTC process from demonstration-scale trials conducted at the 10kt facility at Invica Industries, using feedstocks with similar solids fraction, reaction temperature, and residence time, are applied in this study. Following the HTC process, PC is assessed based on previous modelling work (Farthing 2020). The energy consumption data for both HTC and PC are validated through comparisons with previous studies (Design et al. 2017; Bevan et al. 2021).

The carbon balance in biochar production from food waste (FW) digestate considers the fate of carbon in both biogenic and plastic fractions. Plastics undergo significant volatilization during high-temperature carbonization processes, resulting in very low char yields. Full oxidation of plastic components occurs by being converted to volatiles during post carbonisation temperatures with the volatiles then being combusted to raise process heat (Esmizadeh et al. 2020). The char yields from common plastics such as polyethylene, polypropylene and polystyrene are low (Lee et al. 2023; Salami et al. 2024) meaning there is a minimum contribution of fossil carbon to the biochar. Therefore, it is assumed that all plastic content in the received digestate is completely converted to CO_2_. By accounting for complete conversion of fossil carbon in plastic to CO_2_ in our model, we assessed the net biogenic carbon sequestration in our results. The emissions from plastic oxidation during the HTC-PC process were identical to that from incineration. The emissions related to the plastic content are discussed in the Sect. 3.2 discussing the GHG emissions from the process.

#### Carbon sequestration and permanence

Biochar stability is crucial for its role as a long-term climate mitigation tool (Yang et al. 2021). For biochar to qualify as a CDR tool, it must meet environmental standards such as those from the European Biochar Certificate (EBC), which include low levels of heavy metals and organic pollutants like PAHs, as well as evidence of carbon stability over centuries. Several methods can assess the permanence of biochar. Given the challenges of monitoring biochar in soil over time, stability is often evaluated through structural characteristics. Two key techniques for this are the atomic H/C ratio and Stable Polyaromatic Carbon (SPAC) fraction. The H/C ratio is widely used, with an H/C ratio of < 0.7 indicating high stability (EBC 2023). This measure reflects the carbonization and aromaticity of biochar, which are critical for long-term durability and carbon sequestration (Hung et al. 2017; Song et al. 2021).

SPAC, defined as the carbon fraction isolated by hydrogen pyrolysis (HyPy), is another reliable indicator of biochar stability (McBeath et al. 2015; Schmidt et al. 2022). SPAC consists of aromatic clusters with more than seven rings, which resist oxidation and degradation (Meredith et al. 2012, 2017). In contrast, smaller aromatic structures are more susceptible to microbial breakdown (Kanaly and Harayama 2000). Biochar produced by high-temperature pyrolysis, like those in the HyPy method, contains these larger, more stable aromatic rings (Dai et al. 2020; Song et al. 2021). While the Random Reflectance (Ro) method has potential and offers valuable insights into the geochemical stability of biochar, it is not yet included in the EBC standards and is less widely used (Petersen et al. 2023; Chiaramonti et al. 2024; Sanei et al. 2024). This LCA-TEA study focuses on the H/C ratio and SPAC due to their proven reliability in quantifying the carbonization and stability of biochar, their cost-effectiveness, and their alignment with EBC standards (Crombie et al. 2013; EBC 2023; Adhikari et al. 2024).

#### Biochar application and soil effects

Agricultural application of biochar has the potential to provide additional benefits to carbon sequestration. These include increased primary productivity, improved pH and water-holding capacity, lower N_2_O soil emissions, and decreased NPK fertilizer use (Allohverdi et al. 2021; Lin et al. 2024). Limited data are available to approximate these effects, and effects are heavily dependent on soil characteristics and climate conditions (Blanco-Canqui 2021; Meng et al. 2024). In this work, fertilizer replacement based on the NPK availability in the biochar is the only benefit considered due to the lack of data on other potential impacts (Scenario 3).

Nutrient availability in biochar has been discussed in several studies (Phillips et al. 2022; Castejón-del Pino et al. 2023; Hu et al. 2024) and uncertainty on the availability of these nutrients to crops remains. It is assumed that the stable SPAC fraction does not release its associated nutrient content i.e., only the liable fraction releases NPK. As the biochar degrades the NPK content of the labile fraction becomes available to soil, reducing the required fertiliser inputs. The emission factors related to the displaced NPK fertilizers are presented in Table S1 in the SI. The timescale considered for this benefit is one year, attributing the emissions of displaced NPK in the same year of application, although there is uncertainty of when this would be released. It is assumed that the NPK content is evenly distributed within the biochar. Whilst significant uncertainty remains surrounding NPK release, stability of the labile fraction (Ding et al. 2023; Qiu et al. 2023; Rombola et al. 2023), and distribution on the soil, the inclusion of this scenario adds value to the study by quantifying the hypothetical impact of this consideration on the net GHG emissions of FW digestate biochar.

The spreading of biochar is assumed to be carried out using a 25-ton hydraulic loader and a 30-m-wide spreader attached to a tractor equipped with a mounted plough (Fimaks 2017). The application rate is set at 1 t ha^−1^, ensuring that the biochar is incorporated into the soil rather than simply spread on the surface. To calculate the spreading distance, we considered a square-shaped land area, the spreader width, and a linear application pattern, resulting in a spreading distance of 8.3 km. The application rate of 1 t ha^−1^ reflects the maximum allowable rate in the UK under the low-risk waste position (Environment Agency-GOV.UK 2024).

### Techno-economic analysis

Techno-economic analysis was performed considering the costs associated with utility use, transportation, fixed operation, and annualised capital expenditure (CAPEX) (Table 1), based on the process model presented in Section S.2 of SI. CAPEX and fixed operating costs (Fixed OPEX) of biochar production facility were informed by the 10 kt p.a. demonstration facility, half the capacity of considered in the current study. These costs were also validated using previous process modelling and optimisation work (Farthing 2020). CAPEX is estimated at £9.5 M (CAPEX of 4 × HTC reactors at £1.5 M each at 0.19 t-dry h^−1^, 1 × PC rotary kiln at £3.5 M at 0.36 t-dry h^−1^). The annualized CAPEX was calculated assuming a discount rate of 5% and a plant lifetime of 20 years, in line with estimates by independent analyses (WRAP 2016). Electricity and natural gas costs were based on the projected commercial costs by the Department of Energy Security & Net Zero (DESNZ 2023b) and Department for Business, Energy & Industrial Strategy (BEIS 2017).

Biochar spreading costs were taken as an average from the costs of applying organic material to land by WRAP (WRAP 2016). Digestate and biochar transportations in and out the HTC-PC facility were based on a bulker with 25 t-load capacity, with the cost estimates informed by current transportations of digestate to incineration facilities.

A gate fee of £65 was estimated as the average value of two gate fees: £37 for in-vessel composting (IVC), a lower-cost disposal option suitable for biogenic waste without plastic contamination, and £93 for incineration, a higher-cost route typically used for biogenic waste with plastic contamination (WRAP 2021). While we are not suggesting that digestate could be composted or incinerated, these values serve as reference points for estimating the gate fee in the absence of specific data for digestate processing. This estimation aligns with the median gate fee for FW reported in WRAP’s 2023 report (WRAP 2023b). In practice, the gate fee for FW digestate would depend on how the market for CDR credits and biochar develops in the future, along with the necessary payment to achieve a suitable financial return. Given its significant impact on economics, a range of gate fees is considered in the sensitivity analysis (see Sect. 3.4).

### Sensitivity analysis

A sensitivity analysis was performed to identify the effects of different parameters on the levelized cost of CO_2_ removal in biochar. Table S1 in the SI shows the applied parameter changes in the sensitivity analysis. The effects of gate fee, CAPEX, fixed OPEX, transportations of digestate and biochar and the stability of biochar in terms of the change of SPAC content were evaluated to study the effects on the levelized cost.

## Results

### Characteristics of biochar and permanence

Experimental outcomes of elemental and proximate analyses are given in Table 2 demonstrating the characteristics of FW digestate, hydrochar and biochar. The atomic H/C ratios of the biochar are between 0.1–0.4 (Table 2) which agrees with the EBC’s proposed ratio of < 0.7 for high stability (EBC 2023). The produced biochar quality is within the EBC standards considering the H/C, O/C ratios and the heavy metal contents as shown in Table S2 in SI. Additionally, A SPAC content of 84–92% was obtained in the laboratory and demonstration-scale FW digestate-based biochar, revealing its high long-term stability. In subsequent modelling, we assumed an average SPAC content of 88% to be permanently sequestered in the soils post-application to the field and considered a range from 84 to 92% in the sensitivity analysis. The time period of the degradation of remaining labile carbon fraction is uncertain (Ding et al. 2023; Qiu et al. 2023; Rombola et al. 2023), which can range from days to centuries depending on the biochar properties and the soil environment requiring quantification through extensive field trials. Conservatively, carbon contained in the labile fraction is not considered to contribute to greenhouse gas removals in this study.

**Table 2:** Ultimate and proximate analyses of the FW digestate, HTC hydrochar and biochar from lab and kiln tests

	FW digestate	HTC hydrochar	Lab biochar	Kiln biochar
Ultimate analysis (%)^a^				
C	48.7	69.2 ± 1.0	87.2 ± 3.1	90.9 ± 1.7
H	6.5	6.9 ± 0.5	3.1 ± 0.8	1.1 ± 0.1
N	2.9	3.4 ± < 0.1	3.1 ± 0.3	2.0 ± < 0.1
S	–	1.3 ± < 0.1	1.2 ± < 0.1	1.4 ± < 0.1
O^b^	41.9	19.2 ± 1.3	5.5 ± 2.6	4.6 ± 1.8
Proximate analysis				
Volatiles (%)^a^		79.0 ± 0.5	26.7 ± 0.7	25.6 ± 0.9
Fixed C (%)^a^		21.3 ± 0.5	73.3 ± 0.7	74.4 ± 0.9
C yield (%)^c^		–	43.5 ± 1.5	41.5
Ash (wt% dry)	16.2	19.2 ± 0.8	39.4 ± 0.6	41.3 ± 4.4
H/C^d^		1.2 ± 0.09	0.4 ± < 0.1	0.1 ± < 0.1
SPAC (%)		–	92	84
Porosity (%)^e^		51.9	72.8	–
Bulk density (g cm^−3^)		0.71	0.53	–
Skeletal density (g cm^−3^)		1.47	1.94	1.96
BET SA (m^2^ g^−1^)^f^		12.0	217.3	181.9

^a^Dry ash free base

^b^Oxygen determination by difference

^c^Carbon yield calculated on a dry ash free basis from ultimate analysis C Wt. %

^d^H/C on an atomic weight and dry ash free basis from the ultimate analysis

^e^% porosity = (1 − (Bulk/Skeletal)) × 100

^f^BET SA = specific surface area using the BET model at a relative pressure (P/Po) 0.02–0.05

**Fig. 2:**
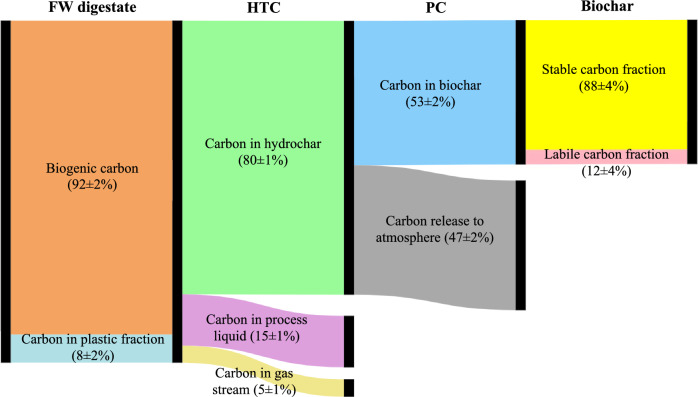
Carbon balance in the system

The carbon balance in the FW digestate-based biochar production is displayed in Fig. 2. The FW digestate consists of 92% of biogenic carbon and 8% carbon from the plastic content. The hydrochar produced by HTC contained approximately 80% of the initial carbon content of the digestate, with 15% leaving in the process liquid and the remaining 5% lost in a CO_2_ rich gas stream (> 95% CO_2_). The PC process captured approximately 53% of the carbon recovered from the HTC process, with the remaining 47% released as volatiles, combusted for energy recovery. The fossil carbon, represented by plastics in the digestate, is assumed to undergo complete oxidation, as outlined in Sect. 2.1.4. This assumption implies that all carbon from plastic components is converted into CO_2_. Consequently, the resulting biochar predominantly consists of biogenic carbon, which originates from organic sources within the digestate. This approach ensures that the biochar effectively sequesters biogenic carbon while mitigating emissions associated with fossil carbon from plastics, aligning with the overall carbon balance strategy discussed in the study. Overall, the HTC-PC process sequestered approximately 42% of the initial carbon from the solids in the FW digestate. The carbon is present in the biochar in labile and stable fractions. For the purposes of carbon sequestration only the stable fraction is considered owing to the temporary nature of the labile fraction in the biochar.

Understanding local resource availability is important to identify the feasibility of the biochar industry in the UK. Meeting the SDG target (FAO 2023) of reducing FW by 50% per capita relative to 2007 by 2030 was applied to estimate the FW availability. This corresponds to 7.7 Mt p.a. of FW and, assuming all FW goes to AD, 1.11 Mt p.a. of digestate generation in 2030. To process this produced FW digestate, 56 HTC-PC facilities of 20 kt p.a. capacity would be required.

### Net GHG emissions of biochar

**Fig. 3:**
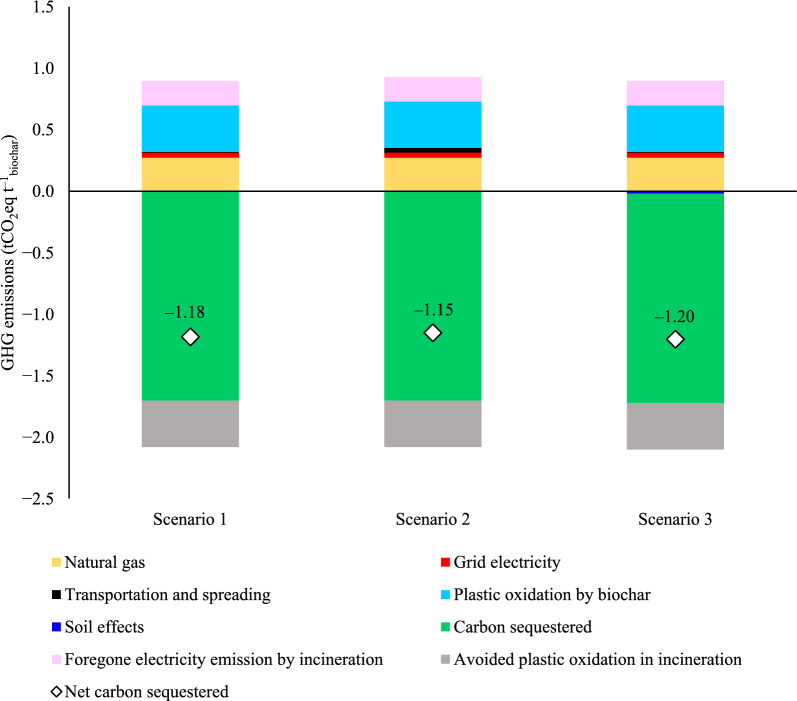
Carbon sequestration potential of HTC-PC process

All the considered scenarios of biochar production from FW digestate achieved substantial net sequestration of atmospheric GHGs (Fig. 3), due to high carbon conversion to biochar (~ 42% of feedstock carbon) and the permanent physical storage of biogenic carbon in biochar (88% SPAC, 1.7 tCO_2_eq t^−1^_biochar_). Net GHG sequestration is reduced by emission sources during biochar production and application, ranging between − 1.18 tCO_2_eq t^−1^_biochar_ and − 1.20 tCO_2_eq t^−1^_biochar_, demonstrating a significant greenhouse gas removal potential across all considered scenarios. The findings are consistent with Hamedani et al. (2019), who conducted an LCA of biochar production using the IMPACT 2002+ and CML LCIA methods. Their study reported GHG removals of − 2.1 tCO₂eq t^−1^ for willow-based biochar and − 0.5 tCO₂eq t^−1^ for manure-based biochar from pyrolysis (Hamedani et al. 2019). Roy et al. (2020) similarly found GHG removals of − 0.5 tCO₂eq t^−1^ for hydrochar from a peat-miscanthus mix (Roy et al. 2020). These results highlight the variability in sequestration potential depending on feedstock and production methods. Hamedani et al. (2019) observed higher GHG removal for willow-based biochar compared to this study, which is expected to be due to the higher carbon and lignin content in wood. In contrast, digestate-derived biochar offers advantages such as reducing methane emissions from raw digestate and serving as a nutrient-rich fertilizer. Compared to manure-based biochar reported by Hamedani et al. (2019) and peat-miscanthus hydrochar reported by Roy et al. (2020), the GHG removal by digestate-based biochar in this study appears superior, likely due to differences in the initial carbon content of digestate and manure. Hydrochar typically has a lower carbon sequestration potential than biochar (de Jager et al. 2022), but the post-carbonization step used to convert hydrochar to biochar in this study helps increase its sequestration potential in comparison with the outcomes of Roy et al. (2020).

The greatest GHG emissions source results from the oxidation of the plastic content of digestate during the HTC and PC processes, and the corresponding release of constituent fossil carbon as CO_2_. Since plastics within the digestate are assumed to undergo complete oxidation during the HTC-PC process, as detailed in Sect. 2.1.4, the resultant biochar primarily contains biogenic carbon. Biogenic carbon originates from organic sources present within the digestate, such as FW or other organic materials. Notably, plastic oxidation emissions are equal to the equivalent release of fossil CO_2_ during FW digestate incineration, as is current practice. However, diverting FW digestate from incineration forgoes renewable electricity generation, resulting in a modest GHG emissions source as UK grid average electricity is no longer displaced by this output. On balance, however, the current incineration of digestate is a net GHG emission source: GHG emissions benefits of displacing UK grid average electricity are calculated as 0.03 tCO_2_eq t^−1^_digestate_ compared with direct emissions of 0.04 tCO_2_eq t^−1^_digestate_ arising from combustion of the plastic fraction. Looking forward, anticipated reduction in the GHG intensity of the UK grid would further increase this net emission source balance.

Energy inputs to biochar production are minimised by operating an integrated HTC and PC processes. Natural gas input to biochar production is significant, contributing 0.27 tCO_2_eq t^−1^_biochar_, whereas electricity inputs to the processes are only a minor emission source. Interestingly, transport of FW digestate in digestate transport scenario (Scenario 2) makes a small contribution to net GHG emissions, despite being significant for biochar production costs, which is discussed in Sect. 3.3.

Co-benefits of biochar application to soil are highly uncertain, and emission reductions associated with soil effects in this work are based on the limited available data (Scenario 3). Scenario 3 assumes that the NPK content of the labile fraction of biochar is available to plants and thus displaces fertiliser production. The corresponding GHG emissions credit for avoiding NPK fertiliser application in Scenario 3 is modest, < 2% of the net GHG removal. Although data are limited, biochar application on agricultural land could realise additional co-benefits related to crop yield, N_2_O emissions mitigation, and water management. The implications of these additional co-benefits are discussed in the discussion section (Sect. 4.3).

### Cost of biochar production

**Fig. 4:**
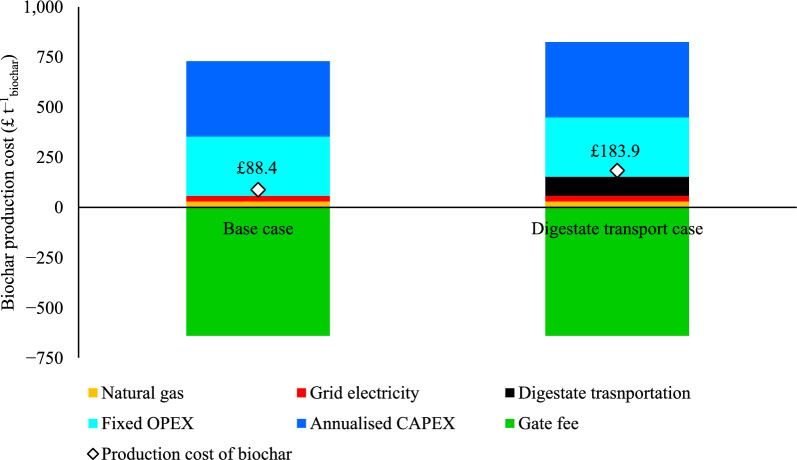
Production cost of 1 tonne of biochar

Biochar production from FW digestate can be achieved at £88 t^−1^_biochar_ in the base case scenario (Scenario 1), with production costs dominated by annualised CAPEX and fixed OPEX being balanced with gate fee revenue received for treating FW digestate. Receiving a gate fee was found to be essential to the financial viability of biochar production from FW digestate. A nominal gate fee of £65 t^−1^_digestate_ is included in results (Fig. 4), equivalent to approximately £641 t^−1^_biochar_. This was selected as the mid-point between compositing (£37 per tonne) and incineration (£93 per tonne). However, owing to its importance and the uncertainty of the gate fee that could be levied in practice, the sensitivity of the biochar production cost to the gate fee is presented in the sensitivity analysis (Sect. 3.4).

The cost of biochar production is highly sensitive to transport distances due to the high moisture content in FW digestate (Fig. 4). In Scenario 2, the biochar production cost increased to £183.9 per tonne when digestate transportation was included. This represents more than double the production cost of Scenario 1, highlighting the significant impact of transportation on overall costs and underscoring the importance of co-locating production facilities with feedstock sources. By contrast, utility costs contribute only a small fraction of the overall production expenses, even though natural gas, as previously discussed (Sect. 3.2), is a significant source of GHG emissions. Scenario 3 was excluded from cost comparisons since it is identical to Scenario 1 up to the biochar production stage.

Haeldermans et al. (2020) reported a biochar production cost range of £362–716 per tonne for various residue streams using conventional pyrolysis (Haeldermans et al. 2020). Similarly, Nematian et al. (2021) identified a wider cost range of £352–1460 per tonne for biochar production from orchard biomass through pyrolysis (Nematian et al. 2021). The comparatively lower production costs observed in this study highlight the significant influence of factors such as feedstock type, production method, and geographical location on overall biochar economics.

### Levelized cost and sensitivity analysis

**Fig. 5:**
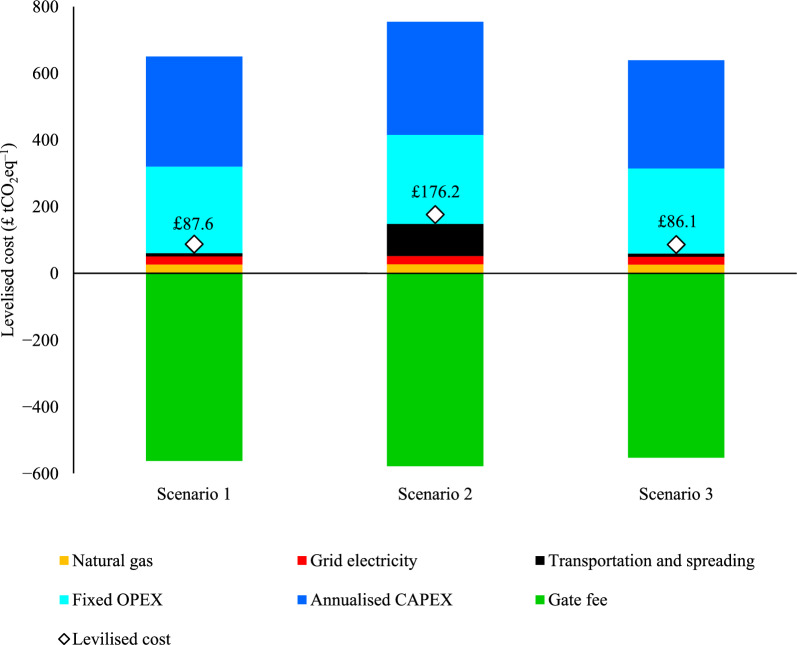
Levelized cost of CO_2_ equivalent avoided for HTC-PC process

All scenarios except Scenario 2 (digestate transport case) achieve commercial scale production with a levelized cost of GHG removal under £100 tCO_2_eq^−1^ (Fig. 5). Differences in the levelized cost of GHG removal are driven by the biochar production cost and net CO_2_ sequestration in the considered scenarios. The scenario representing soil benefits of applying biochar with reducing the fertilizers application (Scenario 3) displays marginal effects associated to the base case, reducing the cost of GHG removal only marginally by £1.5 tCO_2_eq^−1^. Since the uncertainty of labile carbon fraction permanency is very high, further studies on the labile carbon fraction permanency are essential in determining the potential impacts. Potential sequestration of carbon in the labile fraction (12%) of biochar is not considered in these calculations.

**Fig. 6:**
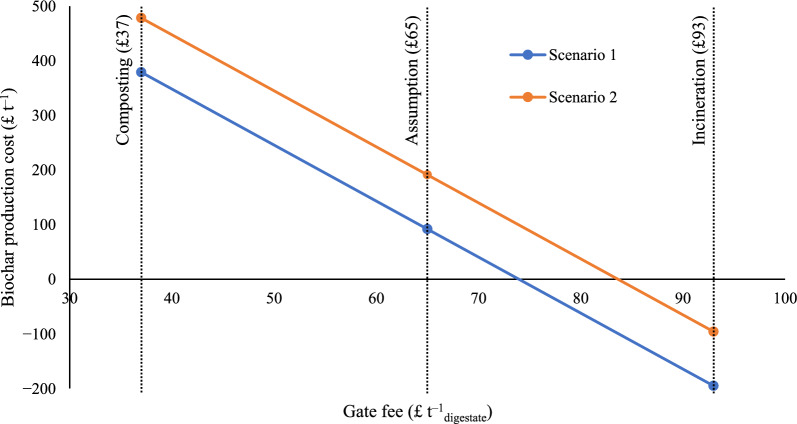
Gate fee effect on biochar production cost

**Fig. 7:**
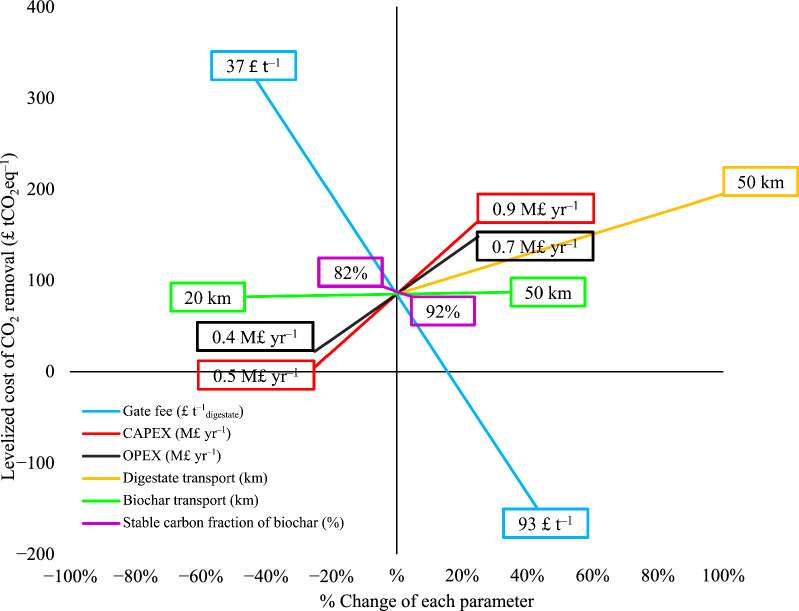
Sensitivity of levelized cost of CO_2_ removal to key parameters

The sensitivity of the results to key production and economic parameters were investigated with respect to their impact on biochar production cost (Fig. 6) and levelized cost (Fig. 7). Sensitivity parameters considered are gate fee, CAPEX, fixed OPEX, transportations of digestate and biochar and the stable fraction of biochar. Varying gate fee within the range of £37 (median for composting) and £93 (median for incineration) (WRAP 2021) was considered. Negative break-even biochar production costs, and hence negative levelized costs of GHG removals, can be achieved with gate fees > £74 t^−1^_digestate_ in Scenario 1 with on-site biochar production and from > £84 t^−1^_digestate_ in Scenario 2 with digestate transport demand (Fig. 6). Without a gate fee, biochar production costs of £759 t^−1^_biochar_ and £858 t^−1^_biochar_ would be realised for Scenarios 1 and 2, respectively, evidencing the importance of charging a gate-fee for commercial viability.

Figure 7 displays changes in levelized cost of CO_2_ removal for Scenario 1 with respect to the varied parameters. Changing gate fee from £37 to £93 t^−1^_digestate_ significantly reduced the levelized cost from £320 to −£150 tCO_2_eq^−1^. Substantial fluctuations in levelized cost were observed with ± 25% change in CAPEX (£5–165 tCO_2_eq^−1^) and fixed OPEX (£22–148 tCO_2_eq^−1^). Transportation and fossil fuel-related costs had only minor influence on the levelized cost. Changing biochar transport distance between 20–50 km displayed negligible changes in the levelized cost. In contrast, varying digestate transport distance from 0–50 km changes levelized cost from £88–£195 tCO_2_eq^−1^_,_ attributed to the higher mass of the wet digestate in comparison to biochar. This finding further emphasises the importance of co-location of AD and HTC-PC facilities, preventing the need to transport high moisture content FW digestate. The change of the stable fraction of the biochar considerably influenced the levelized cost. Increasing the stability by 5% (92% SPAC) dropped the levelized cost by 6%, while decreasing the stability by 5% (84% SPAC) raised the levelized cost by 7%.

## Discussion

### Food waste digestate for carbon sequestration

The conversion of FW digestate to biochar through HTC-PC was found to be a cost-effective method for managing this challenging waste stream that is competitive with other CDR technologies. As noted in previous studies, since the available land area for growing biomass is becoming scarce, biochar production from biowastes such as digestate is timely and appealing (Buss et al. 2019). The availability of wastes, however, can be limited. This is particularly prominent in the case of FWs where there are strong pressures to make more efficient use of food and minimise waste generation. This study shows that for the UK, if the SDG 12.3 target is met, biochar production from FW digestate can achieve an upper limit of GHG removal of 0.18 MtCO_2_eq p.a. in 2030, assuming all FW is sent to AD and the produced digestate is used for biochar production. However, if current FW generation rate per capita is maintained and future FW availability follows the rate of population growth (Office for National Statistics 2023) which is obtained based on extrapolating trends form previous years, then FW availability will increase to 10.1 MT p.a. Using all this FW in AD and the corresponding digestate for biochar production will result in a potential CDR of 0.24 MtCO_2_eq p.a.

The UK government estimates biochar deployment by 2030 to deliver 0–1.1 MtCO_2_eq p.a. removals (Simon et al. 2021), of which this study suggests FW digestate-derived biochar could contribute up to 17–22%. FW digestate-derived biochar therefore represents an important immediate opportunity to establish the potential for biochar CDR in UK towards the 2030 goal. While feedstock availability inherently limits the scale-up potential of FW digestate biochar, this route can help to establish the biochar sector, underpinning future deployments that make use of a variety of feedstocks to scale-up activity towards longer term ambitions of up to 15 MtCO_2_eq p.a. by 2050 (Simon et al. 2021). Globally, FW generation of 1.05 billion-t in 2022 (Forbes et al. 2024) could be utilised to remove 25 MtCO_2_eq p.a. through biochar production. In other jurisdictions with regulatory drivers for the diversion of biogenic wastes from landfill, similar opportunities may exist for biochar production from FW as those identified in the UK market.

### Financial viability of food waste digestate-based biochar

Along with the CDR potential, accurately estimating the financial viability of biochar is needed. Feedstock cost is a critical component in biochar supply chain and deterministic for financial viability, as feedstock alone can contribute for 45–75% of total biochar production cost (Pokharel and Comer 2021). Lower gate fees may be expected for other wet biowastes such as green waste, which could increase overall biochar production cost and pose challenging economics for this application. For example, composting of green waste has a median gate fee of £49 t^−1^ (WRAP 2021), which is below the assumed gate fee here for plastic contaminated FW digestate. Purpose grown or harvested biomass sources, such as woody biomass, are characterised by feedstock costs and unlikely to be compatible for biochar production at costs similar to those found here for FW digestate. Current softwood costs are around £190 t^−1^ (Forest Research 2022), which results in feedstock costs alone reaching £730 t^−1^_biochar_, equivalent to approximately £420 tCO_2_eq^−1^ sequestered if relating with digestate-based biochar. Competition with other biomass uses, including other CDR technologies such as bioenergy with carbon capture and storage (BECCS), may ultimately limit the availability of feedstock for biochar production. Development of frameworks to enable location-specific comparative analysis of competing uses of inherently limited waste and biomass sources is needed to support decision making in this area.

A gate fee > £74 t^−1^_digestate_ makes biochar production from FW digestate favourable achieving the break-even production cost of biochar. This is less than current gate fee for incineration (£93 t^−1^_digestate_) that FW AD operators potentially pay to dispose FW digestate. Moving towards biochar production can therefore provide financial benefit for FW AD digesters. However, biochar being an emerging product, it’s market value as a CDR product and expected co-benefits from soil application are highly uncertain. Current market prices of biochar reported are as high as £1000–£5000 t^−1^ (OxfordBiochar 2024; Soilfixer 2024; WoodlandBiochar 2024). Identified biochar production cost in this work comes < £200 t^−1^ even in the worst case scenario, providing clear evidence of potential high profitability should reported market values be achieved in reality. Current biochar market prices are not in line with its value solely as a CDR: at £1000 t^−1^_biochar_, FW digestate biochar would exhibit a levelized cost of CO_2_ removal of £820 tCO_2_eq^−1^, whereas 2024 puro earth CORC biochar price index (CORCCHAR) is significantly lower of £114.63 per tCO_2_eq removal (138.21 Euro tCO_2_eq^−1^ removal) (puro.earth 2024).

The price of carbon heavily dictates the economic viability of CDR technologies, including biowaste-to-biochar. A greenhouse gas removal report created by Royal Society and Royal Academy of Engineering (The Royal Society and Royal Academy of Engineering 2018) indicates that most CDR methods require a projected price for carbon of £100 tCO_2_eq^−1^ to become economically feasible. The present study shows FW digestate biochar can achieve a carbon price of < £100 tCO_2_eq^−1^ avoided, with a gate fee of over £64 t^−1^_digestate_ indicating its commercial viability. In terms of competing CDR technologies, direct air carbon capture and storage (DACCS) and enhanced weathering are estimated at the highest cost (£900 tCO_2_eq^−1^), while BECCS (£50–270 tCO_2_eq^−1^) and biochar (£14–130 tCO_2_eq^−1^) are at the lower end. The key to achieving levelized costs less than £100 tCO_2_eq^−1^ is the ability to charge a gate fee for the received FW digestate. The primary benefit with the application of AD digestate towards biochar production is avoiding the current considerable cost for the incineration.

### Biochar agriculture field application

Biochar applied to agricultural soils offers various potential benefits, such as improved crop yield, water retention, and reduced greenhouse gas emissions (e.g., N_2_O). It enhances soil NPK dynamics by reducing nitrogen losses, improving phosphorus availability in acidic soils, and increasing potassium retention through its high cation exchange capacity (CEC). These benefits make biochar a valuable tool for sustainable agriculture and nutrient management (Jindo et al. 2020).

This study focuses on the long-term sequestration of stable fractions of biochar (88%), which is critical for its role in CDR. However, the decomposition dynamics of labile carbon fractions are also important, as they affect overall carbon sequestration and nutrient availability. While labile carbon decomposes more slowly than raw organic matter, studies show that its persistence varies. For instance, labile carbon content dropped from 26 to 9% within two years in vineyard soils (Rombolà et al. 2023), and the mean residence time of labile carbon is estimated at 108 days (Wang et al. 2016). With this uncertainty, this study justifies the consideration of the immediate availability of NPK and its marginal effect on GHG removal and cost benefits, by accounting for only (10%) the labile fraction.

Biochar production also creates by-products like HTC liquid, which could be used if it meets UK standards. However, the long-term effects of biochar on temperate UK soils remain uncertain due to limited studies. More research is needed to assess the environmental impact, soil emissions, and long-term stability for large-scale applications of biochar. Establishing biochar standards will be crucial for its role in achieving the UK’s greenhouse gas removal goals, and its growing availability from biowaste offers a promising solution.

## Conclusions

The performed LCA-TEA on FW digestate based biochar production and its soil application in this work identified its high potential as a CDR technology. The economic viability was identified with the obtained low GHG mitigation costs of < £100 tCO_2_eq^−1^ (125 USD tCO_2_eq^−1^) by minimising the digestate transport through co-location of the biochar production with AD facilities. With the considered 88% stable fraction in this work, biochar stores 1.7 tCO_2_eq t^−1^ effectively removing atmospheric greenhouse gases, achieving net emissions of 1.15–1.20 tCO_2_eq t^−1^_biochar_. A number of 28 biochar facilities of scales 20 kt p.a. will be needed to process 50% of the UK’s projected available FW digestate by 2030, sequestering 93 ktCO_2_eq p.a., The gate fee charged to process FW digestate influenced heavily on establishing the economic success of biochar production from FW digestate. GHG removals cost becomes negligible with gate fees > £74 t^−1^_digestate_ with on-site biochar production and with gate fees > £84 t^−1^_digestate_ with digestate transportation. Further studies on the potential technology enhancements to reduce fossil-fuel use and the investigation of the co-benefits of agricultural applications of biochar are required to establish FW to biochar scheme.

## Supplementary Information

Supplementary Material 1.

## Data Availability

The authors declare that the data supporting the findings of this study are available within the paper and supplementary information files.

## Abbreviations

AD: Anaerobic digestate
HTC: Hydrothermal carbonisation
PC: Post carbonisation
CDR: Carbon dioxide removal
GHG: Greenhouse gas
FW: Food waste
LCA: Life cycle assessment
TEA: Techno-economic assessment
EBC: European biochar certificate
SDG: Sustainable development goals
PET: Polyethylene terephthalate
HDPE: High density polyethylene
PP: Polypropylene
LDPE: Low density polyethylene
CAPEX: Capital expenditure
OPEX: Operational expenditure
NG: Natural gas
BET SA: Brunauer–Emmett–Teller Surface Area
BSI PAS: British Standard Institution’s Publicly Available Specification
BEIS: Department for Business, Energy, and Industrial Strategy
WRAP: Waste and Resources Action Programme

## References

[CR1] Adhikari S, Moon E, Paz-Ferreiro J, Timms W (2024) Comparative analysis of biochar carbon stability methods and implications for carbon credits. Sci Total Environ 914:169607. 10.1016/j.scitotenv.2023.169607 38154640 10.1016/j.scitotenv.2023.169607

[CR2] Allohverdi T, Mohanty AK, Roy P, Misra M (2021) A review on current status of biochar uses in agriculture. Molecules. 10.3390/molecules26185584 10.3390/molecules26185584 PMC8470807 34577054

[CR3] Aragón-Briceño C, Ross AB, Camargo-Valero MA (2017) Evaluation and comparison of product yields and bio-methane potential in sewage digestate following hydrothermal treatment. Appl Energy 208:1357–1369. 10.1016/j.apenergy.2017.09.019

[CR4] Aragón-Briceño CI, Grasham O, Ross AB et al (2020) Hydrothermal carbonization of sewage digestate at wastewater treatment works: influence of solid loading on characteristics of hydrochar, process water and plant energetics. Renew Energy 157:959–973. 10.1016/j.renene.2020.05.021

[CR5] Asibor JO, Clough PT, Nabavi SA, Manovic V (2021) Assessment of optimal conditions for the performance of greenhouse gas removal methods. J Environ Manage. 10.1016/j.jenvman.2021.113039 10.1016/j.jenvman.2021.113039 34153633

[CR6] Aspray TJ, Tompkins D (2019) Plastic in food waste at compost sites. In: Scottish Environ. Prot. Agency. https://bbia.org.uk/wp-content/uploads/2019/11/plastic-in-compost-SEPA-report-2019.pdf. Accessed 10 May 2023

[CR7] Aspray TJ, Dimambro M, Eco C (2018) Investigation into plastic in food waste derived digestate and soil. https://www.researchgate.net/publication/324910012_Investigation_into_plastic_in_food_waste_derived_digestate_and_soil#fullTextFileContent. Accessed 8 Dec 2023

[CR8] BEIS (2017) Updated Energy and emissions projections 2016: Annex M: Growth assumptions and prices. In: Dep. Energy Secur. Net Zero Dep. Business, Energy Ind. Strateg. https://www.gov.uk/government/publications/updated-energy-and-emissions-projections-2016

[CR9] Bevan E, Fu J, Zheng Y (2021) RSC advances challenges and opportunities of hydrothermal carbonisation in the UK ; case study in Chirnside. 34870–34897. 10.1039/d1ra06736b 10.1039/d1ra06736b PMC9042953 35494736

[CR10] Blanco-Canqui H (2021) Does biochar improve all soil ecosystem services? GCB Bioenergy 13:291–304. 10.1111/gcbb.12783

[CR11] Buss W, Jansson S, Wurzer C, Mašek O (2019) Synergies between BECCS and biochar—maximizing carbon sequestration potential by recycling wood ash. ACS Sustain Chem Eng 7:4204–4209. 10.1021/acssuschemeng.8b05871

[CR599] Castejón-del Pino R, Cayuela ML, Sánchez-García M, Sánchez-Monedero MA (2023) Nitrogen availability in biochar-based fertilizers depending on activation treatment and nitrogen source. Waste Manag 158:76–83. 10.1016/j.wasman.2023.01.007 10.1016/j.wasman.2023.01.007 36641823

[CR12] Cavali M, Libardi Junior N, de Mohedano RA et al (2022) Biochar and hydrochar in the context of anaerobic digestion for a circular approach: an overview. Sci Total Environ 822:153614. 10.1016/j.scitotenv.2022.153614 35124030 10.1016/j.scitotenv.2022.153614

[CR13] Chen C, Liang W, Fan F, Wang C (2021) The effect of temperature on the properties of hydrochars obtained by hydrothermal carbonization of waste camellia oleifera shells. ACS Omega. 10.1021/acsomega.1c01787 10.1021/acsomega.1c01787 PMC8246692 34235326

[CR14] Chen L, Zhu X, Zheng Y et al (2024) Development of high-strength lightweight concrete by utilizing food waste digestate based biochar aggregate. Constr Build Mater 411:134142. 10.1016/j.conbuildmat.2023.134142

[CR15] Chiaramonti D, Lehmann J, Berruti F et al (2024) Biochar is a long-lived form of carbon removal, making evidence-based CDR projects possible. Biochar. 10.1007/s42773-024-00366-7

[CR17] Crombie K, Mašek O, Sohi SP et al (2013) The effect of pyrolysis conditions on biochar stability as determined by three methods. GCB Bioenergy 5:122–131. 10.1111/gcbb.12030

[CR18] Dai Y, Wang W, Lu L et al (2020) Utilization of biochar for the removal of nitrogen and phosphorus. J Clean Prod 257:120573. 10.1016/j.jclepro.2020.120573

[CR19] de Jager M, Schröter F, Wark M, Giani L (2022) The stability of carbon from a maize-derived hydrochar as a function of fractionation and hydrothermal carbonization temperature in a Podzol. Biochar. 10.1007/s42773-022-00175-w

[CR20] de la Rubia MA, Villamil JA, Rodriguez JJ, Mohedano AF (2018) Effect of inoculum source and initial concentration on the anaerobic digestion of the liquid fraction from hydrothermal carbonisation of sewage sludge. Renew Energy 127:697–704. 10.1016/j.renene.2018.05.002

[CR21] DEFRA (2013) Incineration of Municipal Solid Waste. In: Dep. Environ. Food Rural Aff. https://assets.publishing.service.gov.uk/government/uploads/system/uploads/attachment_data/file/221036/pb13889-incineration-municipal-waste.pdf. Accessed 2 Feb 2023

[CR22] DEFRA (2018) 25 Year environment plan progress report. In: Dep. Environ. Food Rural Aff. https://assets.publishing.service.gov.uk/government/uploads/system/uploads/attachment_data/file/803266/25yep-progress-report-2019-corrected.pdf. Accessed 2 Feb 2023

[CR23] DEFRA (2020) Department for environment, food & rural affairs—British survey of fertiliser practice: fertiliser use on farm for the 2019 crop year

[CR24] DEFRA (2021) Official Statistics Section 3: Anaerobic digestion. https://www.gov.uk/government/statistics/area-of-crops-grown-for-bioenergy-in-england-and-the-uk-2008-2020/section-3-anaerobic-digestion. Accessed 22 Feb 2024

[CR25] DEFRA (2024) Anaerobic digestion National statistics. In: Dep. Environ. Food Rural Aff. https://www.gov.uk/government/statistics/farm-practices-survey-february-2023-greenhouse-gas-mitigation/anaerobic-digestion. Accessed 14 May 2024

[CR26] Deng X, Teng F, Chen M et al (2024) Exploring negative emission potential of biochar to achieve carbon neutrality goal in China. Nat Commun. 10.1038/s41467-024-45314-y 10.1038/s41467-024-45314-y PMC10844326 38316787

[CR27] Department for Environment F& RA (2020) Household food waste to be collected separately by 2023 and 50,000 city trees to be planted in urban tree challenge fund. In: Defra Press Off. https://deframedia.blog.gov.uk/2020/02/10/household-food-waste-to-be-collected-separately-by-2023-and-50000-city-trees-to-be-planted-in-urban-tree-challenge-fund/

[CR28] Design P, Efficiency E, Lucian M, Fiori L (2017) Hydrothermal carbonization of waste biomass: cost analysis. Energies. 10.3390/en10020211

[CR29] DESNZ (2023a) Data tables 1 to 19: supporting the toolkit and the guidance in Green Book supplementary guidance: valuation of energy use and greenhouse gas emissions for appraisal. DESNZ

[CR30] DESNZ (2023b) Energy and emissions projections 2022 to 2040: Annex M: Growth assumptions and prices. In: Dep. Energy Secur. Net Zero. https://www.gov.uk/government/publications/energy-and-emissions-projections-2022-to-2040. Accessed 10 Jan 2024

[CR31] Ding Y, Liu Y, Liu S et al (2016) Biochar to improve soil fertility. A review. Agron Sustain Dev. 10.1007/s13593-016-0372-z

[CR32] Ding X, Li G, Zhao X et al (2023) Biochar application significantly increases soil organic carbon under conservation tillage: an 11-year field experiment. Biochar. 10.1007/s42773-023-00226-w

[CR33] Directorate-General for Internal Market, Industry E and Sme (2022) Commission adopts new rules further developing the Fertilising Products Regulation. In: Eur. Comm. https://single-market-economy.ec.europa.eu/news/commission-adopts-new-rules-further-developing-fertilising-products-regulation-2022-03-22_en

[CR34] EBC (2012-2023) European Biochar Certificate - Guidelines for a Sustainable Production of Biochar. Carbon Standards International (CSI), Frick, Switzerland. (https://european-biochar.org). Version 10.3 from 5th Apr 2022

[CR35] Environment Agency (2014) Quality Protocol Anaerobic digestate End of waste criteria for the production and use of quality outputs from anaerobic digestion of source-segregated biodegradable waste. Environment Agency, London

[CR36] Environment Agency-GOV.UK (2024) Storing and spreading biochar to benefit land: LRWP 61. https://www.gov.uk/government/publications/low-risk-waste-positions-landspreading/storing-and-spreading-biochar-to-benefit-land-lrwp-61. Accessed 30 Nov 2024

[CR37] Esmizadeh E, Tzoganakis C, Mekonnen TH (2020) Degradation behavior of polypropylene during reprocessing and its biocomposites: thermal and oxidative degradation kinetics. Polymers (Basel). 10.3390/POLYM12081627 10.3390/polym12081627 PMC7464851 32707872

[CR500] European Parliament and Council of the European Union (2019) Regulation (EU) 2019/1009 laying down rules on the making available on the market of EU fertilising products. https://eur-lex.europa.eu/eli/reg/2019/1009/oj. Accessed 10 Dec 2024

[CR38] FAO (2023) Food and Agriculture Organization of the United Nations. https://www.fao.org/sustainable-development-goals-data-portal/data/indicators/1231-global-food-losses/en. Accessed 23 Nov 2023

[CR39] Farthing S (2020) Hydrothermal carbonisation of digestate: An investigation into the technical design, economic feasibility and future potential of the process. the University of Nottingham

[CR40] Ferrentino R, Merzari F, Fiori L, Andreottola G (2020) Coupling hydrothermal carbonization with anaerobic digestion for sewage sludge treatment: Influence of HTC liquor and hydrochar on biomethane production. Energies. 10.3390/en13236262

[CR41] Fimaks (2017) MANURE SPREADER 25 TONS. http://fimaks.com/en/manure-spreader/25-tons-manure-spreader-fmgr-25/. Accessed 5 Sept 2023

[CR42] Forbes H, Peacock E, Abbot N, Jones M (2024) Food Waste Index Report 2024. think eat save: tracking progress to halve global food waste. In: United Nations Environ. Program. https://wedocs.unep.org/20.500.11822/45230. Accessed 10 Apr 2024

[CR43] Forest Research (2022) Tools and Resources Timber Price Indices. https://www.forestresearch.gov.uk/tools-and-resources/statistics/statistics-by-topic/timber-statistics/timber-price-indices/. Accessed 10 Dec 2022

[CR44] Forster P, Storelvmo T, Armour K et al (2021) The Earth’s energy budget, climate feedbacks, and climate sensitivity. In: Masson-Delmotte, Zhai VP, Pirani A (eds) Climate change: the physical science basis. Cambridge University Press, Cambridge, pp 923–1054

[CR45] Gao Y, Shao G, Yang Z et al (2021) Influences of soil and biochar properties and amount of biochar and fertilizer on the performance of biochar in improving plant photosynthetic rate: a meta-analysis. Eur J Agron 130:126345. 10.1016/j.eja.2021.126345

[CR46] Goglio P, Williams AG, Balta-Ozkan N et al (2020) Advances and challenges of life cycle assessment (LCA) of greenhouse gas removal technologies to fight climate changes. J Clean Prod. 10.1016/j.jclepro.2019.118896

[CR47] GOV.UK (2022) Environmental taxes, reliefs and schemes for businesses. https://www.gov.uk/green-taxes-and-reliefs. Accessed 10 Oct 2022

[CR48] Haeldermans T, Campion L, Kuppens T et al (2020) A comparative techno-economic assessment of biochar production from different residue streams using conventional and microwave pyrolysis. Bioresour Technol 318:124083. 10.1016/j.biortech.2020.124083 32916464 10.1016/j.biortech.2020.124083

[CR49] Hamedani SR, Kuppens T, Malina R et al (2019) Life cycle assessment and environmental valuation of biochar production: two case studies in Belgium. Energies 12:1–21. 10.3390/en12112166

[CR50] Houses of Parliament (2010) Biochar Post Note. House Parliam, pp 1–4

[CR600] Hu W, Zhang Y, Rong X, et al (2024) Biochar and organic fertilizer applications enhance soil functional microbial abundance and agroecosystem multifunctionality. Biochar 6. 10.1007/s42773-023-00296-w

[CR51] Hung C-Y, Tsai W-T, Chen J-W et al (2017) Characterization of biochar prepared from biogas digestate. Waste Manag 66:53–60. 10.1016/j.wasman.2017.04.034 28487174 10.1016/j.wasman.2017.04.034

[CR52] ISO Compliance Register (2024) The Separation of Waste (England) Regulations 2024. https://isocomplianceregister.co.uk/iso_article/the-separation-of-waste-england-regulations-2024/

[CR53] Jindo K, Audette Y, Higashikawa FS et al (2020) Role of biochar in promoting circular economy in the agriculture sector. Part 1: a review of the biochar roles in soil N, P and K cycles. Chem Biol Technol Agric 7:1–12. 10.1186/s40538-020-00182-8

[CR54] Kanaly RA, Harayama S (2000) Biodegradation of high-molecular-weight polycyclic aromatic hydrocarbons by bacteria. J Bacteriol 182:2059–2067. 10.1128/JB.182.8.2059-2067.2000 10735846 10.1128/jb.182.8.2059-2067.2000 PMC111252

[CR55] Kossińska N, Krzyżyńska R, Ghazal H, Jouhara H (2023) Hydrothermal carbonisation of sewage sludge and resulting biofuels as a sustainable energy source. Energy. 10.1016/j.energy.2023.127337

[CR56] Kumar S, Ankaram S (2019) Chapter 12—waste-to-energy model/tool presentation. Curr Dev Biotechnol Bioeng. 10.1016/B978-0-444-64083-3.00012-9

[CR57] Lee S, Kim YT, Lin KYA, Lee J (2023) Plastic-waste-derived char as an additive for epoxy composite. Materials (Basel). 10.3390/ma16072602 10.3390/ma16072602 PMC10095672 37048896

[CR58] Lefebvre D, Williams A, Kirk GJD et al (2021) An anticipatory life cycle assessment of the use of biochar from sugarcane residues as a greenhouse gas removal technology J Clean. Prod. 10.1016/j.jclepro.2021.127764

[CR59] Li Y, Yao N, Liang J et al (2022) Optimum biochar application rate for peak economic benefit of sugar beet in Xinjiang, China. Agric Water Manag. 10.1016/j.agwat.2022.107880

[CR60] Lin F, Wang H, Shaghaleh H et al (2024) Effects of biochar amendment on N2O emissions from soils with different pH levels. Atmosphere (Basel) 15:4–15. 10.3390/atmos15010068

[CR61] Mahmud R, Moni SM, High K, Carbajales-Dale M (2021) Integration of techno-economic analysis and life cycle assessment for sustainable process design—a review. J Clean Prod. 10.1016/j.jclepro.2021.128247

[CR62] Marzeddu S, Cappelli A, Ambrosio A et al (2021) A life cycle assessment of an energy-biochar chain involving a gasification plant in Italy. Land. 10.3390/land10111256

[CR63] Matuštík J, Hnátková T, Kočí V (2020) Life cycle assessment of biochar-to-soil systems: a review. J Clean Prod. 10.1016/j.jclepro.2020.120998

[CR64] McBeath AV, Wurster CM, Bird MI (2015) Influence of feedstock properties and pyrolysis conditions on biochar carbon stability as determined by hydrogen pyrolysis. Biomass Bioenerg 73:155–173. 10.1016/j.biombioe.2014.12.022

[CR65] Meng X, Zheng E, Hou D et al (2024) The effect of biochar types on carbon cycles in farmland soils: a meta analysis. Sci Total Environ. 10.1016/j.scitotenv.2024.172623 10.1016/j.scitotenv.2024.172623 38653414

[CR66] Meredith W, Ascough PL, Bird MI et al (2012) Assessment of hydropyrolysis as a method for the quantification of black carbon using standard reference materials. Geochim Cosmochim Acta 97:131–147. 10.1016/j.gca.2012.08.037

[CR67] Meredith W, McBeath A, Ascough P, Bird M (2017) Analysis of biochars by hydropyrolysis (HyPy). In: Singh B, Camps-Arbestain MLJ (eds) Biochar: a guide to analytical methods. CSIRO Publishing, pp 187–198

[CR68] Murillo HA, Pagés-Díaz J, Díaz-Robles LA et al (2022) Valorization of oat husk by hydrothermal carbonization: optimization of process parameters and anaerobic digestion of spent liquors. Bioresour Technol 343:126112. 10.1016/j.biortech.2021.126112 34648962 10.1016/j.biortech.2021.126112

[CR69] Musa U, Castro-Díaz M, Uguna CN, Snape CE (2022) Effect of process variables on producing biocoals by hydrothermal carbonisation of pine Kraft lignin at low temperatures. Fuel 325:124784. 10.1016/j.fuel.2022.124784

[CR70] Nasrollahzadeh M, Nezafat Z, Shafiei N (2021) Lignin chemistry and valorization. In: Nezafat Z (ed) Biopolymer-Based Metal Nanoparticle Chemistry for Sustainable Applications. Elsevier, pp 145–183

[CR71] Nematian M, Keske C, Ng’ombe JN (2021) A techno-economic analysis of biochar production and the bioeconomy for orchard biomass. Waste Manag 135:467–477. 10.1016/j.wasman.2021.09.014 34626931 10.1016/j.wasman.2021.09.014

[CR72] Nicholas A, Hussein M, Zainal Z, Khadiran T (2019) Chapter 12—activated carbon for shape-stabilized phase change material. Synth Technol Appl Carbon Nanomater. 10.1016/B978-0-12-815757-2.00013-9

[CR73] Office for National Statistics (2023) United Kingdom population mid-year estimate. https://www.ons.gov.uk/peoplepopulationandcommunity/populationandmigration/populationestimates/timeseries/ukpop/pop. Accessed 2 Aug 2023

[CR74] Oni BA, Oziegbe O, Olawole OO (2019) Significance of biochar application to the environment and economy. Ann Agric Sci 64:222–236. 10.1016/j.aoas.2019.12.006

[CR75] OxfordBiochar (2024) Biochar. https://www.oxfordbiochar.org/. Accessed 27 Jan 2024

[CR76] Paul S, Dutta A, Defersha F (2018) Biocarbon, biomethane and biofertilizer from corn residue: a hybrid thermo-chemical and biochemical approach. Energy 165:370–384. 10.1016/j.energy.2018.09.182

[CR77] Petersen HI, Lassen L, Rudra A et al (2023) Carbon stability and morphotype composition of biochars from feedstocks in the Mekong Delta, Vietnam. Int J Coal Geol. 10.1016/j.coal.2023.104233

[CR598] Phillips CL, Meyer KM, Garcia-Jaramillo M, et al (2022) Towards predicting biochar impacts on plant-available soil nitrogen content. Biochar 4:1–15. 10.1007/s42773-022-00137-2

[CR78] Pokharel R, Comer B (2021) Economics of biochar. https://www.canr.msu.edu/news/economics-of-biochar. Accessed 10 Oct 2022

[CR79] puro.earth (2024) CORC CARBON REMOVAL INDEXES. https://puro.earth/corc-carbon-removal-indexes. Accessed 3 Dec 2024

[CR80] Qiu H, Hu Z, Liu J et al (2023) Effect of biochar on labile organic carbon fractions and soil carbon pool management index. Agronomy 13:1–15

[CR81] Rombolà AG, Greggio N, Fabbri D et al (2023) Changes of labile, stable and water-soluble fractions of biochar after two years in a vineyard soil. Environ Sci Adv 2:1587–1599. 10.1039/d3va00197k

[CR82] Roy P, Dutta A, Gallant J (2020) Evaluation of the life cycle of hydrothermally carbonized biomass for energy and horticulture application. Renew Sustain Energy Rev 132:110046. 10.1016/j.rser.2020.110046

[CR83] Salami L, Patinvoh RJ, Taherzadeh MJ (2024) Waste plastic char as adsorbent for removal of pollutants from landfill leachates–a critical review. Environ Adv 16:100522. 10.1016/j.envadv.2024.100522

[CR84] Sanei H, Rudra A, Przyswitt ZMM et al (2024) Assessing biochar’s permanence: an inertinite benchmark. Int J Coal Geol. 10.1016/j.coal.2023.104409

[CR85] Schmidt HP, Abiven S, Hagemann N, Drewer JM zu (2022) Permanace of soil applied biochar: An executive summary for Global Biochar Carbon Sink certification. Biochar J 69–74. https://doi.org/www.biochar-journal.org/en/ct/109

[CR800] Shackley S, Ibarrola Esteinou R, Hopkins D, Hammond J (2014) Biochar Quality Mandate (BQM) version 1.0. https://www.semanticscholar.org/paper/bd9efaa4a34bcfbfa616891ef87dbe670f761ecc

[CR87] Simon R, Mitchell A, Evans C, et al (2021) Greenhouse gas removal methods and their potential UK deployment. In: A Rep. Dep. Business, Energy Ind. Strateg. by Elem. Energy UK Cent. Ecol. Hydrol. https://assets.publishing.service.gov.uk/government/uploads/system/uploads/attachment_data/file/1026988/ggr-methods-potential-deployment.pdf

[CR88] Soilfixer (2024) Biochar. https://www.soilfixer.co.uk/. Accessed 27 Jan 2024

[CR89] Song S, Lim JW, Lee JTE et al (2021) Food-waste anaerobic digestate as a fertilizer: The agronomic properties of untreated digestate and biochar-filtered digestate residue. Waste Manag 136:143–152. 10.1016/j.wasman.2021.10.011 34666296 10.1016/j.wasman.2021.10.011

[CR90] Stirling RJ, Snape CE, Meredith W (2018) The impact of hydrothermal carbonisation on the char reactivity of biomass. Fuel Process Technol 177:152–158. 10.1016/j.fuproc.2018.04.023

[CR91] Suarez E, Tobajas M, Mohedano AF et al (2023) Effect of garden and park waste hydrochar and biochar in soil application: a comparative study. Biomass Convers Biorefinery. 10.1007/s13399-023-04015-0

[CR92] Svoboda K, Baxter D, Martinec J (2006) Nitrous oxide emissions from waste incineration. Chem Pap 60:78–90. 10.2478/s11696-006-0016-x

[CR93] Szwaja S, Magdziarz A, Zajemska M et al (2019) Investigation on thermal decomposition of biogas digestate to producer gas. IOP Conf Ser Earth Environ Sci. 10.1088/1755-1315/214/1/012140

[CR94] Terlouw T, Bauer C, Rosa L, Mazzotti M (2021) Life cycle assessment of carbon dioxide removal technologies: a critical review. Energy Environ Sci 14:1701–1721. 10.1039/d0ee03757e

[CR95] The Royal Society, Royal Academy of Engineering (2018) Greenhouse gas removal. https://royalsociety.org/-/media/policy/projects/greenhouse-gas-removal/royal-society-greenhouse-gas-removal-report-2018.pdf

[CR96] Victor L, Moves W (2020) AD & Composting Market Survey Report. In: Waste Resour. Action Plan. https://www.r-e-a.net/wp-content/uploads/2021/01/AD-Composting-Market-Survey-Report.pdf

[CR97] Wang J, Xiong Z, Kuzyakov Y (2016) Biochar stability in soil: Meta-analysis of decomposition and priming effects. GCB Bioenergy 8:512–523. 10.1111/gcbb.12266

[CR98] Wang B, Fu H, Han L et al (2021) Physicochemical properties of aged hydrochar in a rice-wheat rotation system: a 16-month observation. Environ Pollut 272:116037. 10.1016/j.envpol.2020.116037 33248832 10.1016/j.envpol.2020.116037

[CR99] WoodlandBiochar (2024) Biochar. https://www.woodlandbiochar.co.uk. Accessed 27 Jan 2024

[CR100] WRAP (2015) Organics recycling industry status report 2015. In: Waste Resour. Action Plan. https://www.nnfcc.co.uk/files/mydocs/asori 2015.pdf. Accessed 10 May 2023

[CR101] WRAP (2016) Digestate and compost use in agriculture. In: Waste Resour. Action Plan. https://wrap.org.uk/sites/default/files/2020-08/WRAP-Digestate-compost-good-practice-guide-reference-version.pdf. Accessed 5 Oct 2023

[CR102] WRAP (2018) Composition of plastic waste collected via kerbside. In: Waste Resour. Action Plan. https://wrap.org.uk/sites/default/files/2020-10/WRAP-Composition of Plastic Waste Collected via Kerbside v2.pdf. Accessed 2 Oct 2023

[CR103] WRAP (2021) Gate Fees 2019/20 Report: Comparing the costs of alternative waste treatment options. In: Waste Resour. Action Plan. https://wrap.org.uk/sites/default/files/2021-01/Gate-Fees-Report-2019-20.pdf. Accessed 2 Aug 2023

[CR104] WRAP (2022) Review: Technologies to Optimise the Value of Digestate (2020). In: Waste Resour. Action Plan. https://www.r-e-a.net/wp-content/uploads/2021/01/Review-Technologies-to-Optimise-the-Value-of-Digestate.pdf. Accessed 10 May 2023

[CR105] WRAP (2023a) UK Food Waste & Food Surplus – Key Facts. In: Waste Resour. Action Plan. https://wrap.org.uk/sites/default/files/2023-11/WRAP-Food-Surplus-and-Waste-in-the-UK-Key-Facts-Nov-2023.pdf. Accessed 3 May 2024

[CR106] WRAP (2023b) Plastics in composts and digestates. In: Waste Resour. Action Plan. https://www.wrap.ngo/resources/report/plastics-composts-and-digestates. Accessed 6 Mar 2024

[CR107] Xiao H, Zhang D, Tang Z et al (2022) Comparative environmental and economic life cycle assessment of dry and wet anaerobic digestion for treating food waste and biogas digestate. J Clean Prod. 10.1016/j.jclepro.2022.130674

[CR108] Yang F, Zuo X, Zhou Y et al (2021) Stability of biochar in five soils: Effects from soil property. Environ Prog Sustain Energy. 10.1002/ep.13775

[CR109] Yoganandham ST, Sathyamoorthy G, Renuka RR (2020) Chapter 8—Emerging extraction techniques: Hydrothermal processing. Sustain Seaweed Technol. 10.1016/B978-0-12-817943-7.00007-X

