# Supplementary material for: Biowaste to biochar: a techno-economic and life cycle assessment of biochar production from food-waste digestate and its agricultural field application
Source: Biochar. 2025 Mar 10;7(1):50. doi: 10.1007/s42773-025-00456-0 (PMC11893672; doi:10.1007/s42773-025-00456-0)
Supplement: Supplementary file 1 — Supplementary Material 1. [file 42773_2025_456_MOESM1_ESM.docx]

**Title:** Biowaste to Biochar: A techno-economic and life cycle assessment of biochar production from food-waste digestate and its agricultural field application

**Authors:**

Disni Gamaralalage^a,^*, Sarah Rodgers^a^, Andrew Gill^b^, Will Meredith^c^, Tom Bott^d^, Helen West^d^, Jessica Alce^e^, Colin Snape^c^, Jon McKechnie^a^

**Affiliations:**

^a^ Sustainable Process Technologies Research Group, Faculty of Engineering, The University of Nottingham, Nottingham, NG7 2RD, UK

^b^ R&D manager, Invica Industries, The University of Nottingham, Energy Technologies Building, Jubilee Campus, Nottingham, NG7 2TU, UK

^c^ Faculty of Engineering, The University of Nottingham, Energy Technologies Building, Jubilee Campus, Nottingham, NG7 2TU, UK

^d^ School of Biosciences, Sutton Bonington Campus, The University of Nottingham, Leicestershire, LE12 5RD, UK

^e^ Strategy & Development, Severn Trent Green Power, The Stables, Radford, Chipping Norton OX7 4EB, UK

*Corresponding author: disni.gamaralalage@nottingham.ac.uk

**Keywords**

biochar, anaerobic digestate, food waste, life cycle assessment, techno-economic assessment, greenhouse gas removal

**Statements and Declarations**

The authors have no relevant financial or non-financial interests to disclose and the information regarding the sources of funding are provided at the end of the manuscript. The authors declare that the data supporting the findings of this study are available within the paper and supplementary information files.

**Author contributions**

Conceptualization: Jon McKechnie, Disni Gamaralalage, Sarah Rodgers; Methodology: Jon McKechnie, Disni Gamaralalage; Formal analysis and investigation: Disni Gamaralalage, Sarah Rodgers; Writing - original draft preparation: Disni Gamaralalage; Writing - review and editing: Jon McKechnie, Disni Gamaralalage, Sarah Rodgers, Will Meredith, Tom Bott, Helen West, Colin Snape, Andrew Gill, Jessica Alce; Funding acquisition: Colin Snape; Resources: Andrew Gill, Jessica Alce, Tom Bott, Will Meredith, Helen West; Supervision: Jon McKechnie.

**Article Highlights**

· Plastic contamination makes food waste digestate expensive to manage

· Hydrothermal carbonisation combined with post carbonisation is an efficient route to produce stable biochar

· Distributed biochar production sequesters carbon for less than £100 per tCO_2_eq

**Abbreviations:**

AD: anaerobic digestate, HTC: hydrothermal carbonisation, PC: post carbonisation, CDR: carbon dioxide removal , GHG: greenhouse gas, FW: food waste, LCA: life cycle assessment, TEA: techno-economic assessment, EBC: European biochar certificate, SDG: Sustainable development goals, PET: polyethylene terephthalate, HDPE: high density polyethylene, PP: polypropylene, LDPE: low density polyethylene, CAPEX:, capital expenditure, OPEX: operational expenditure, NG: natural gas, BET SA: Brunauer-Emmett-Teller Surface Area, BSI PAS:, British Standard Institution’s Publicly Available Specification, BEIS: Department for Business, Energy, and Industrial Strategy, WRAP: Waste and Resources Action Programme

**Funding Sources:**

This work was supported by the Biotechnology and Biological Sciences Research Council [grant number BB/V011596/1] and the Department of Business, Energy and Industrial Strategy through the Direct Air Capture and Greenhouse Gas Removal Programme Phase 1 for the grant "Bio-waste to Biochar (B to B) via Hydrothermal Carbonisation and Post-Carbonisation”

*(*[*https://www.gov.uk/government/publications/direct-air-capture-and-other-greenhouse-gas-removal-technologies-competition/projects-selected-for-phase-1-of-the-direct-air-capture-and-greenhouse-gas-removal-programme*](https://www.gov.uk/government/publications/direct-air-capture-and-other-greenhouse-gas-removal-technologies-competition/projects-selected-for-phase-1-of-the-direct-air-capture-and-greenhouse-gas-removal-programme)*).*

**Supplementary Information**

*Table S1: Parameters applied in the study*

| **Parameter** | **Value** | **Unit** |
| --- | --- | --- |
| FW digestate |  |  |
| Digestate moisture content | 70 | % |
| Nominal gate fee | 65 | £ t^–1^_digestate_ |
| Digestate transport cost | 0.22 | £ tkm^–1^ |
| Composition of the 5% plastic fraction^a^ |  |  |
| PET | 40.3 | % |
| HDPE | 21.6 | % |
| PP | 10.2 | % |
| Film (assumed LDPE) | 15.7 | % |
| Other plastics | 12.2 | % |
| HTC-PC |  |  |
| Natural gas emission factor^b^ | 0.183 | kgCO_2_ kWh^–1^ |
| Natural gas cost^c^ | 21.1 | £ MWh^–1^ |
| Grid electricity emission factor^b^ | 0.085 | kgCO_2_ kWh^–1^ |
| Grid electricity cost^d^ | 55.8 | £ MWh^–1^ |
| Biochar yield (%) | 32.5 | wt% dry digestate |
| Carbon content in biochar (%) | 52.8 | wt% dry biochar |
| Biochar soil application |  |  |
| Overall inorganic N fertilizer application rate, as N^e^ | 0.09 | tonnes |
| Overall inorganic P fertilizer application rate, as P_2_O_5_^e^ | 0.02 | tonnes |
| Overall inorganic K fertilizer application rate, as K_2_O^e^ | 0.02 | tonnes |
| Inorganic N fertilizer, as N emission factor | 4.622 | kgCO_2_ kg^–1^ |
| Inorganic P fertilizer, as P_2_O_5_ emission factor | 1.953 | kgCO_2_ kg^–1^ |
| Inorganic K fertilizer, as K_2_O emission factor | 2.11 | kgCO_2_ kg^–1^ |
| Digestate Incineration |  |  |
| Digestate LHV (dry basis, including plastic content)^f^ | 16.1 | MJ kg^–1^ |
| Latent heat of vaporisation of water | 2.26 | MJ kg^–1^ |
| Electricity conversion efficiency^g^ | 27 | % |
| Sensitivity Analysis |  |  |
| Gate fee for in-vessel composting^h^ | 37 | £ t^–1^_digestate_ |
| Gate fee for incineration^h^ | 93 | £ t^–1^_digestate_ |
| CAPEX | ±25 | % |
| Fixed OPEX | ±25 | % |
| Digestate transport | 0-50 | km |
| Biochar transport | 20-50 | km |

^a^(WRAP 2018), ^b^(DESNZ 2023a), ^c^(BEIS 2017), ^d^(DESNZ 2023b), ^e^(DEFRA 2020), ^f^(Szwaja et al. 2019), ^i^(DEFRA 2013), ^h^(WRAP 2021)

S1. Experimental investigation of the characteristics, production, and permanence of biochar

In this work, biochar is produced from FW digestate using HTC and PC, where an intermediate product of hydrochar is produced after the HTC process. HTC process is conducted in a demonstration facility located at Invica Industries site in Immingham, while the PC is conducted as lab-scale and demonstration-scale kiln trials.

S1.1 Characteristics of FW digestate, hydrochar and biochar

The characteristics of the feedstock (FW digestate) and the produced hydrochar and biochar are evaluated by performing elemental analysis (EA) and proximate analysis. EA was conducted with a CHNS analyser (Leco Instruments). Approximately 50 mg of sample was used to determine CHN under combustion at 950 °C in 100% O_2_, while 250 mg of sample was used for S analysis at 1350 °C. The samples were run in quintuplicate.

Proximate analysis was conducted by thermo-gravimetric analysis (TGA, TA Instruments). 50 mg of sample was weighed onto a platinum pan and heated to 110 °C (10 °C min^–1^) under 100% N_2_ (1 bar, 100 mL min^–1^) and held for 30 minutes to determine moisture content. The sample was then ramped to 950 °C (10 °C min^–1^, 1 bar, 100 mL min^–1^) and held isothermally for 30 mins to determine the amount of volatiles present. The gas was switched to air (1 bar, 100 mL min^–1^) and held isothermally for 30 mins to determine fixed carbon and ash contents. Samples were run in quintuplicate.

S1.2 Biochar production

The conversion of hydrochar to biochar is performed by a PC step. The PC is not a part of the core HTC pilot plant. The PC following the fluidised bed dryer was added for further thermal treatment of the hydrochar to convert into biochar. This type of post-treatments have also been shown to remove organic phytotoxic compounds and therefore could be useful for soil application (Hitzl et al. 2018; Bahcivanji et al. 2020). The lab-scale PC trials were conducted with 30 duplications following the procedure of weighting 7.5 g of the HTC product (>4.75 mm) into a ceramic boat and placing in a horizontal tube furnace. The sample was purged in 100% N_2_ (1 bar, 1 L min^–1^) for 15 minutes to remove air and then carbonised at higher temperatures (550 - 800 ˚C) for 0.5 hours at a ramp rate of 10 ˚C min^–1^ under 100% N_2_ (1 bar, 1 L min^–1^) and held isothermally for 30 minutes. The demonstration-scale kiln trails are conducted at Invica Industries site in Immingham for 25 kg of digestate samples with a PC temperature of 750 ˚C. The 40% w/w yield was consistent with the lab-scale tests.

S1.3 Permanence of biochar

Stable polycyclic aromatic carbon (SPAC) content in the biochar is used to evaluate the stability of biochar to sequester carbon for a long time. SPAC is the percentage of carbon remaining after HyPy analysis and represents the portion of the carbon thought to be stable in the environment over centennial timescales. HyPy performed with heating under a hydrogen pressure of 150 bar and a flow rate of 5 L min^–1^ from ambient to 250 °C at 300 °C min^–1^, and then to 550 °C at 8 °C where it was held for 2 mins (Meredith et al. 2012).

S2. Biochar production by HTC and PC process systems

Fig. S1 represents the process flow considered in this work referring to the HTC pilot plant and the added PC process based on the assumptions for converting hydrochar into biochar (Farthing 2020). The FW digestate is fed through a biomass hopper and then a mixer to form a homogenous feedstock. The feedstock is then pumped, considering the saturation pressure of the products inside the reactor (24 bar). HTC reactor operates under autogenous pressure (saturation pressure of water at the reaction temperature) up to 40 bar at 200 ˚C. The feedstock is preheated before entry to the reactor using a jacketed pipe containing water from the heat recovery line. The remaining heat to raise the feedstock to reaction temperature is provided by a natural gas-powered steam boiler. The mixture of hydrochar and process liquid, exits the reactor via output pipes and is passed through a series of depressurisation tanks. The slurry from the depressurisation tanks then passes through a hydrocyclone for ash reduction. Then a filter press is used to reduce the moisture content of the slurry (around 50%). A fluidised bed dryer is used to dry the filter cake resulting in hydrochar with moisture content about 12.5%. The fluidised bed dryer consists of a bed of filter cake and a mixture of hot process gases and air passing through at a high enough velocity to keep the filter cake in a fluidised state, resulting in high heat transfer and drying rates. A heat recovery system is integrated in the plant using a series of heat exchangers and jacketed pipes containing water at 105 °C, maintained as a liquid under pressure. The system preheats the feedstock, recovers heat from the reaction products and preheats the air to the fluidised bed dryer. The PC is added at the end representing a furnace operates at a temperature of 750 ˚C to convert the dried hydrochar to biochar. The mass and energy balance of the system is given in Fig. S2.


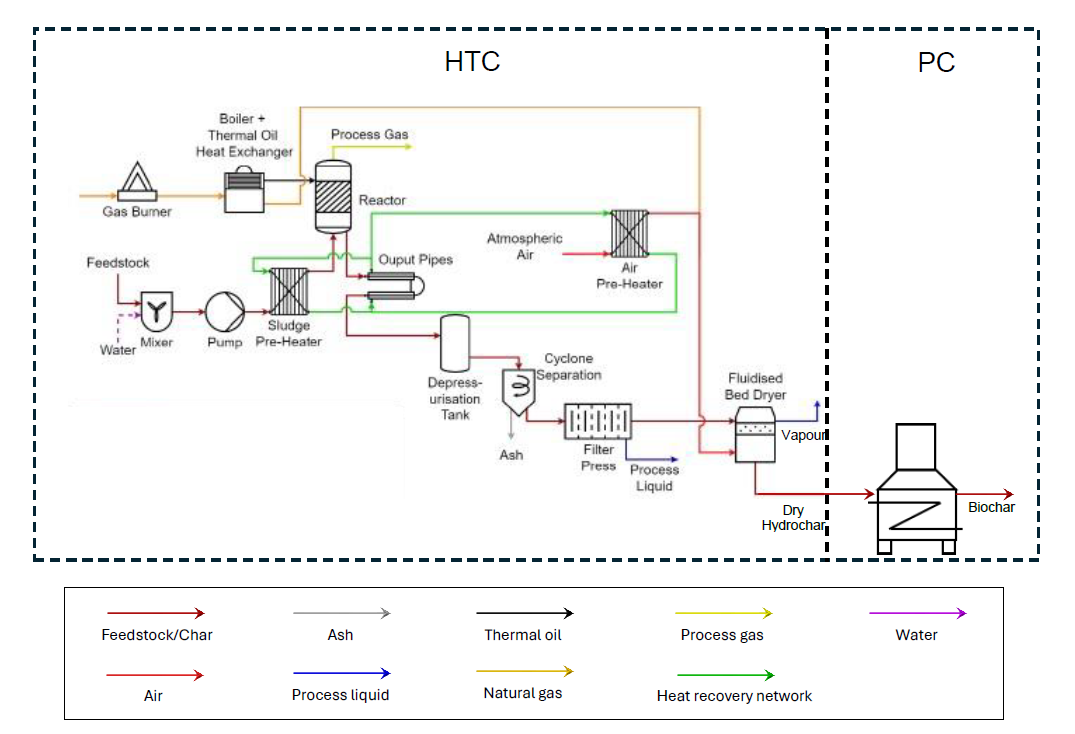


**Fig. S1**: HTC and PC process systems

**PC**

**HTC**

Biochar

(1.0 t h^–1^)


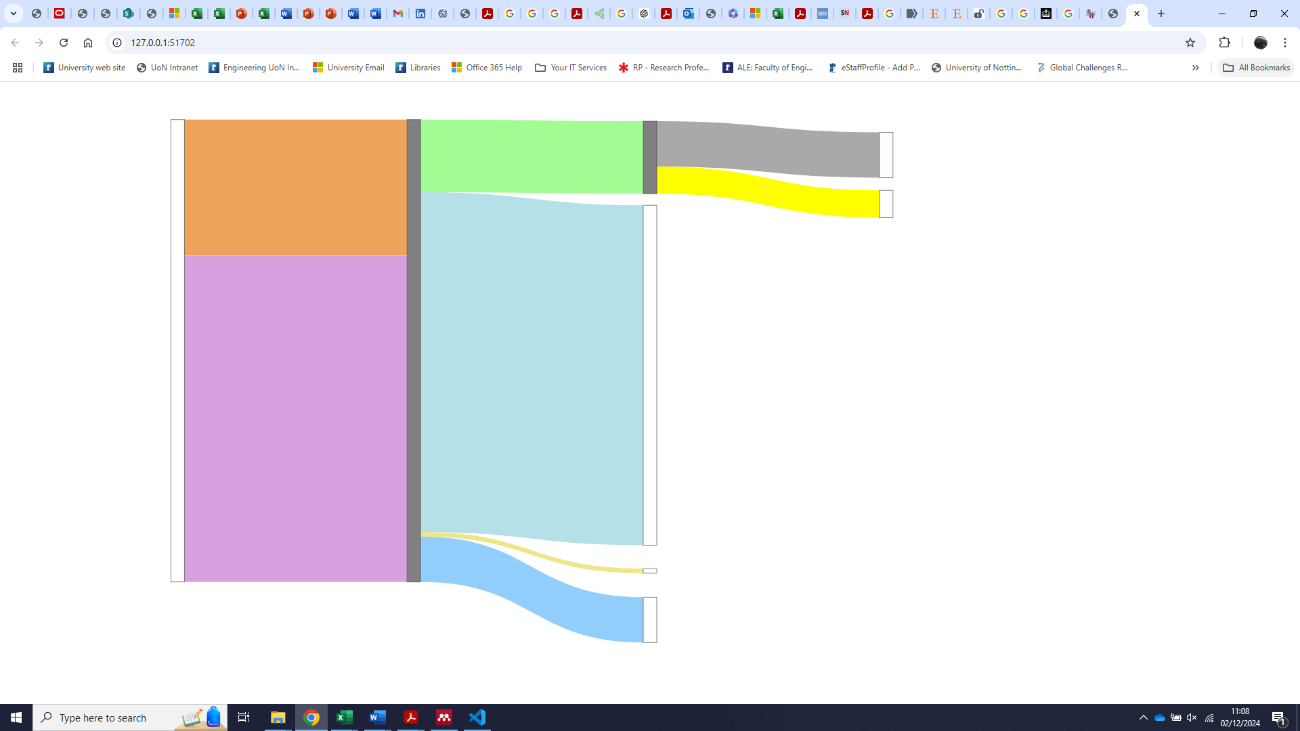


**a**

Water loss

(1.0 t h^–1^)

Syngas

(0.6 t h^–1^)

Process gas

(0.1 t h^–1^)

Process water

(7.5 t h^–1^)

Hydrochar

(1.6 t h^–1^)

Digestate-wet

(7.1 t h^–1^)

Digestate-dry

(3.1 t h^–1^)

**HTC**

**PC**


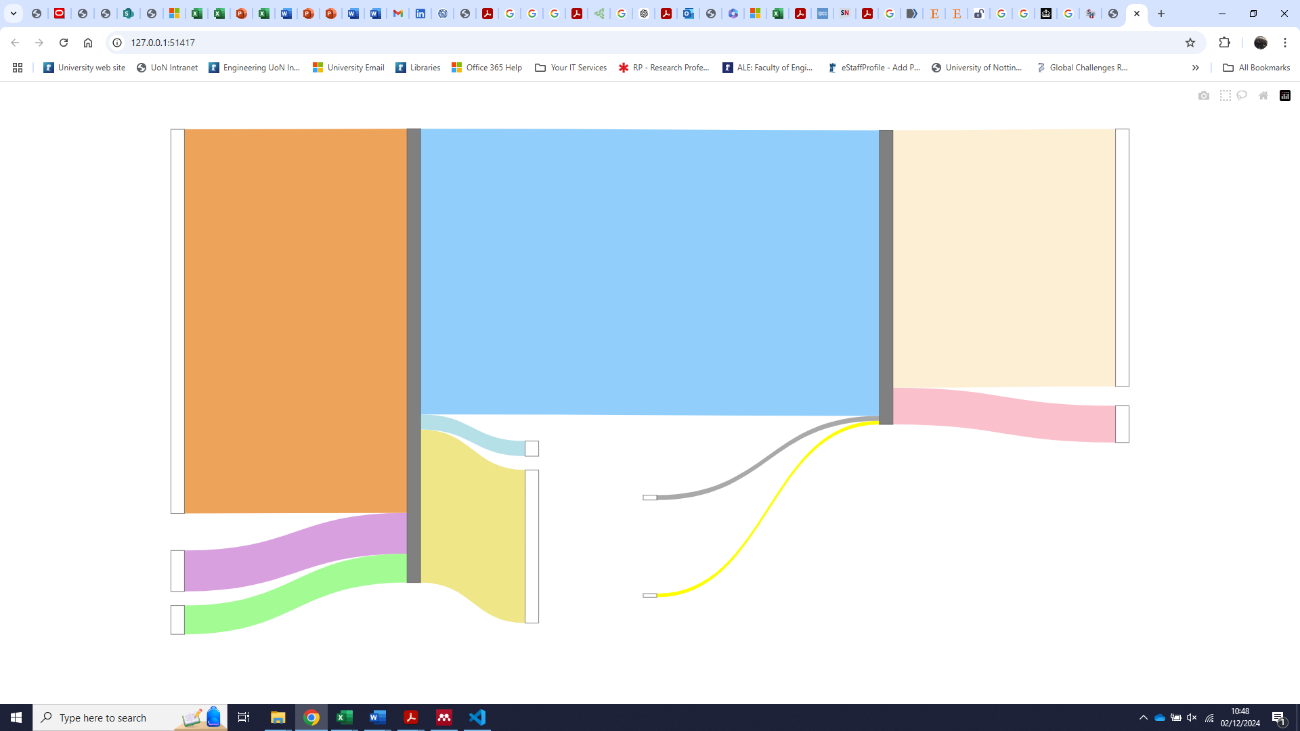


Syngas

(4.702 MJ)

Biochar

(33,000 MJ)

**b**

Liquid

(19,613 MJ)

Gas

(1,941 MJ)

Digestate

(49,231 MJ)

NG

(5,243 MJ)

Electricity

(3,692 MJ)

Hydrochar

(36,612 MJ)

Electricity

(462 MJ)

NG

(628 MJ)

**Fig. S2**: Material (a) and energy (b) balance in the system

S3: Comparison of produced biochar with the European Biochar Certificate (EBC) standards

Table S2 demonstrates the EBC standards for all the EBC classification classes as EBC-Feed Plus, EBC-Feed, EBC-Agro Organic, EBC-Agro, EBC-Urban, EBC-Consumer Materials and EBC-Basic Materials (EBC 2023) and their comparison with the produced digestate-based biochar’s quality considering the H/C ratio, O/C ratio and the presence of heavy metals. The produced digestate-based biochar’s target application is for the soil application which needs to align with the EBC-Agro standard. The produced digestate-based biochar in the study satisfies all the EBC-Agro requirements except slightly higher concentration in copper.

*Table S2: Comparison of produced biochar with EBC standards^a^*

| **EBC -Certification Class** | **EBC-Feed Plus** | **EBC-Feed** | **EBC-Agro Organic** | **EBC-Agro** | **EBC-Urban** | **EBC- Consumer Materials** | **EBC- Basic Materials** | **Biochar** |
| --- | --- | --- | --- | --- | --- | --- | --- | --- |
|  |  |  |  |  |  |  |  |  |
| H / C_org_ | < 0.4 | | < 0.7 | | | | | 0.19 |
| O/ C_org_ | < 0.4 | | | | | | | 0.035 |
| **Heavy metals** | | | | | | | | |
| Pb | 10 g t^–1^ (88% DM) | | 45 g t^–1^DM | 120 g t^–1^DM | | | declaration, no limit values for certification | 16 g t^–1^DM |
| Cd | 0.8 g t^–1^ (88% DM) | | 0.7 g t^–1^DM | 1.5 g t^–1^DM | | |  | <0.2 g t^–1^DM |
| Cu | 70 g t^–1^DM | | | 100 g t^–1^DM | | |  | 120 g t^–1^DM |
| Ni | 25 g t^–1^ DM | | | 50 g t^–1^DM | | |  | 28 g t^–1^DM |
| Hg | 0.1 g t^–1^ (88% DM) | | 0.4 g t^–1^DM | 1 g t^–1^DM | | |  | <0.07 g t^–1^DM |
| Zn | 200 g t^–1^DM | | | 400 g t^–1^DM | | |  | 158 g t^–1^DM |
| Cr | 70 g t^–1^DM | | | 90 g t^–1^DM | | |  | 41 g t^–1^DM |
| As | 2 g t^–1^ (88% DM) | | 13 g t^–1^DM | 13 g t^–1^DM | | |  | 2.3 g t^–1^DM |

DM: Dry mass, ^a^(EBC 2023)

References

Bahcivanji L, Gascó G, Paz-Ferreiro J, Méndez A (2020) The effect of post-pyrolysis treatment on waste biomass derived hydrochar. Waste Manag 106:55–61. https://doi.org/10.1016/j.wasman.2020.03.007

BEIS (2017) Updated Energy and emissions projections 2016: Annex M: Growth assumptions and prices. In: Dep. Energy Secur. Net Zero Dep. Business, Energy Ind. Strateg. https://www.gov.uk/government/publications/updated-energy-and-emissions-projections-2016

DEFRA (2020) Department for Environment, Food & Rural Affairs - British Survey of Fertiliser Practice: Fertiliser use on farm for the 2019 crop year

DEFRA (2013) Incineration of Municipal Solid Waste. In: Dep. Environ. Food Rural Aff. https://assets.publishing.service.gov.uk/government/uploads/system/uploads/attachment_data/file/221036/pb13889-incineration-municipal-waste.pdf. Accessed 2 Feb 2023

DESNZ (2023a) Green Book supplementary guidance: valuation of energy use and greenhouse gas emissions for appraisal: Data-tables-1 to 19: supporting the toolkit and the guidance. In: Dep. Energy Secur. Net Zero. https://assets.publishing.service.gov.uk/media/6567994fcc1ec5000d8eef17/data-tables-1-19.xlsx. Accessed 3 Apr 2024

DESNZ (2023b) Energy and emissions projections 2022 to 2040: Annex M: Growth assumptions and prices. In: Dep. Energy Secur. Net Zero. https://www.gov.uk/government/publications/energy-and-emissions-projections-2022-to-2040. Accessed 10 Jan 2024

EBC (2023) European Biochar Certificate- Guidelines for a sustainable production of biochar. In: Eur. Biochar Certif. https://www.european-biochar.org/media/doc/2/version_en_10_3.pdf

Farthing S (2020) Hydrothermal carbonisation of digestate: An investigation into the technical design, economic feasibility and future potential of the process. the University of Nottingham

Hitzl M, Mendez A, Owsianiak M, Renz M (2018) Making hydrochar suitable for agricultural soil: A thermal treatment to remove organic phytotoxic compounds. J Environ Chem Eng 6:7029–7034. https://doi.org/10.1016/j.jece.2018.10.064

Meredith W, Ascough PL, Bird MI, et al (2012) Assessment of hydropyrolysis as a method for the quantification of black carbon using standard reference materials. Geochim Cosmochim Acta 97:131–147. https://doi.org/10.1016/j.gca.2012.08.037

Szwaja S, Magdziarz A, Zajemska M, et al (2019) Investigation on Thermal Decomposition of Biogas Digestate to Producer Gas. IOP Conf Ser Earth Environ Sci 214:. https://doi.org/10.1088/1755-1315/214/1/012140

WRAP (2018) Composition of plastic waste collected via kerbside. In: Waste Resour. Action Plan. https://wrap.org.uk/sites/default/files/2020-10/WRAP-Composition of Plastic Waste Collected via Kerbside v2.pdf. Accessed 2 Oct 2023

WRAP (2021) Gate Fees 2019/20 Report: Comparing the costs of alternative waste treatment options. In: Waste Resour. Action Plan. https://wrap.org.uk/sites/default/files/2021-01/Gate-Fees-Report-2019-20.pdf. Accessed 2 Aug 2023
